# A ternary association of KRas, EphA2, and integrins controls PDAC cell behavior

**DOI:** 10.1016/j.jbc.2026.113421

**Published:** 2026-08-10

**Authors:** Mahak Fatima, Nasir Haider, Stuart A. Cain, Bipul R. Acharya, Megan R. Chastney, Junzhe Zha, Matthew C. Jones, Stacey Warwood, David Knight, Derek A. O’Reilly, Jonathan D. Humphries, Benjamin T. Goult, Claus Jørgensen, Martin J. Humphries

**Affiliations:** 1 Manchester Cell-Matrix Centre, Faculty of Biology, Medicine and Health, Manchester Academic Health Science Centre, The University of Manchester, Manchester, UK; 2 Cancer Research UK Manchester Institute, The University of Manchester, Manchester, UK; 3 Biological Mass Spectrometry Core Facility, Faculty of Biology, Medicine and Health, The University of Manchester, Manchester, UK; 4 Department of Hepatobiliary and Pancreatic Surgery, Manchester Royal Infirmary, Manchester, UK; 5 Biochemistry, Cell and Systems Biology, Institute of Systems, Molecular and Integrative Biology, University of Liverpool, Liverpool, UK

**Keywords:** pancreatic, cancer, KRas, EphA2, integrins, BioID, desmoplasia, stroma

## Abstract

Pancreatic ductal adenocarcinoma (PDAC) is one of the most lethal cancers, primarily because late detection, a lack of effective therapies and an abundant desmoplastic reaction result in elevated proliferation, invasion, and immune evasion. KRas is the most frequently mutated driver gene in PDAC and, while drugs are now available that target this GTPase, there remains a need for further pharmacological options to treat the disease. As a first step to identify potential targets, this study aimed to define the molecular environment of KRas using proximity proteomics. A KRas^G12V^-biotin ligase chimera was stably expressed in several PDAC cell lines and organoids, and associating proteins identified following biotin addition, streptavidin affinity isolation, and mass spectrometry. Among the 126 proteins that were specifically associated in all cell lines, five integrin subunits and the receptor tyrosine kinase ephrin type-A receptor 2 (EphA2) were consistently enriched. Both classes of receptor have been previously implicated in both Ras signaling and desmoplastic responses. A range of protein biochemical and microscopic analyses confirmed the spatial association of integrin β1 and EphA2 with KRas. Cellular KRas levels were reduced following depletion of both integrin β1 and EphA2, and depletion of EphA2 inhibited KRas-dependent migration. Taken together, these findings highlight a ternary crosstalk between receptor tyrosine kinase, integrin, and KRas signaling, which provides insight into PDAC progression and signposts potential therapeutic vulnerabilities.

Pancreatic cancer (PC) is one of the most lethal malignancies with an overall 5-year survival rate in the order of 10% (1, 2, 3). Pancreatic ductal adenocarcinoma (PDAC) comprises a large majority of PC cases and is driven by a diverse range of mutations (4). The most effective treatment option for early-stage PDAC is currently surgical removal in combination with gemcitabine- and FOLFIRINOX (folinic acid, 5-fluorouracil, irinotecan, and oxaliplatin)-based chemotherapy (5, 6); however, approximately 80% of PDAC cases present at an advanced stage that is beyond the point where these treatments are effective. In addition to delayed diagnosis and a lack of effective therapies (7, 8, 9), a highly developed desmoplastic response contributes to the severity of the disease (10, 11, 12). Stromal rigidity not only drives proliferation and invasion, but also results in an immunosuppressive environment (13).

KRas is the most mutated gene in PDAC, being present in more than 90% of cases (14, 15), and, together with other mutated genes such as CDKN2A, TP53 and SMAD4, is required for the initiation and progression of PDAC (16, 17). KRas is a small GTPase switch that regulates several signaling pathways, including MAP kinase (MAPK) and PI-3-kinase (PI3K)/Akt (3, 18, 19, 20). In cancer, KRas is most frequently mutated at residues 12, 13, and 61, which shifts the molecule to an active, GTP-bound state and hence generates a continuous signaling cascade that drives tumor growth, survival, and development (21). The first KRas inhibitor approved for clinical use targeted the G12 C mutation (22, 23); however, while G12 C is common in lung adenocarcinoma, it has a low frequency in PDAC (24). Additional KRas inhibitors are in development for targeting other mutations, or the ON state of the enzyme, but these are not yet approved (25, 26, 27, 28). Thus, there remains an unmet need to identify new therapeutic targets to inhibit the transmission of oncogenic KRas signaling (29).

Many PDAC tumors have a majority stromal compartment, and collagens are among the most enriched components of the PDAC extracellular matrix (ECM) (30). The ECM is primarily recognized by integrin receptors, which interpret the chemical and mechanical properties of the ECM to control clonogenic growth, apoptosis, migration, and EMT, in part through the MAPK and PI3K/Akt pathways (31, 32). The expression of many integrin subunits correlates negatively with PDAC prognosis (33, 34, 35, 36, 37). The desmoplastic response also contributes to drug resistance and inaccessibility due to the physical barrier imposed by stromal ECM (38), poor vascular circulation and elevated hypoxia (39). Finally, the ECM serves as a reservoir for growth factors and is therefore involved in regulating cellular function *via* other signal transduction receptors (28). In this context, the expression of several receptor tyrosine kinases (RTKs) correlates negatively with outcomes in PDAC, including members of the ephrin receptor family (40, 41, 42, 43). Ephrins and ephrin receptors again activate the PI3K/Akt (44) and MAPK signaling pathways (45) downstream of oncogenic KRas. The role of EphA2 has been extended from original roles in cell-cell repulsion (46) and ligand-dependent inhibition of MAPK and Akt (47) in normal cells to metastasis (45), Tiam1-mediated endocytosis (48), activation of Akt and MAPK activity (49), and ECM deposition in cancer cells (50).

Several studies have elucidated the Ras interactome using different techniques, including proximity biotinylation (BioID) (40, 51, 52, 53, 54, 55, 56). The associations identified were not only limited to the canonical Ras interactors, but also revealed interactions linked to other major signaling receptors including RTKs and integrins (40, 56, 41). However, to date, none of these reports explored the relevance of these interactions. Therefore, to identify potential therapeutic targets in PDAC, this study aimed to build on this earlier work and to obtain a consensus definition of the molecular environment of KRas in a range of PC cell lines using BioID. Among the identified proteins were receptors that regulate diverse signaling responses in cancers, again including integrins and RTKs, in addition to canonical KRas interactors. Subsequently, integrin β1 and EphA2 were confirmed to associate with KRas and to regulate KRas levels. These findings highlight a common crosstalk between RTK signaling, integrin signaling, and KRas signaling.

## Results

### Definition of an oncogenic KRas interactome in PDAC cell lines

To identify proteins associated with oncogenic KRas in PDAC cells, BioID was performed on two disease-representative lines, KP4 and Suit-2. An immortalized normal human pancreatic epithelial cell line (h6c7) was included for comparison. BirA∗ and BirA∗-KRas^G12V^ were cloned into the pCDH-EF1 lentiviral vector with a blue fluorescent protein (BFP) tag, a self-cleaving peptide T2A and an N-terminal Myc tag. Cells stably expressing physiological levels of the constructs were generated by lentiviral transduction followed by cell sorting based on the BFP tag. Western blotting with anti-Myc and anti-Ras confirmed the expression and localization of the protein in the target cell lines (Fig. S1, *A* and *B*). To verify the ability of the mutant BirA∗ ligase to biotinylate proteins in the context of the fusion protein, BirA∗- and BirA∗-KRas-expressing cells were labeled with biotin for 24 h, and fluorophore-conjugated Streptavidin was used to detect biotinylated proteins by Western blotting (Fig. S1*A*). Multiple, distinct sets of bands, including the baits themselves, were detected in both samples. To localize stably expressed KRas^G12V^, immunofluorescence imaging was employed using anti-Myc antibody. Staining was present at the cell periphery in the BirA∗-KRas^G12V^-expressing cells, while diffuse cytoplasmic staining was present in BirA∗-expressing cells (Fig. S1*B*). These results demonstrate the successful integration of BioID constructs and their correct localization.

To define the interactome of oncogenic KRas, a label-free, quantitative MS approach was applied to affinity-captured, proximity-labeled biotinylated proteins. A total of 1939 proteins were reproducibly quantified, and pairwise correlation analysis and principal component analysis (PCA) were performed to assess the reproducibility and variance among samples. PCA revealed a distinct separation between the bait and control group for each replicate and highlighting the cell context-dependent nature of the Kras G12 V interactome (Fig. S1*C*). Pairwise comparisons of the replicates indicated a high similarity within the replicates for each bait with a strong Pearson correlation score within the same sample type (Fig. S1*D*). Overall, these analyses identified high similarity between replicates of the same sample type and variance among sample groups, *i.e.*, between BirA∗ and BirA∗-KRas^G12V^. Furthermore, comparisons of ion intensities following filtration and normalization remained unchanged, indicating greater similarity between replicates (Fig. S1*E*).

**Figure 1:**
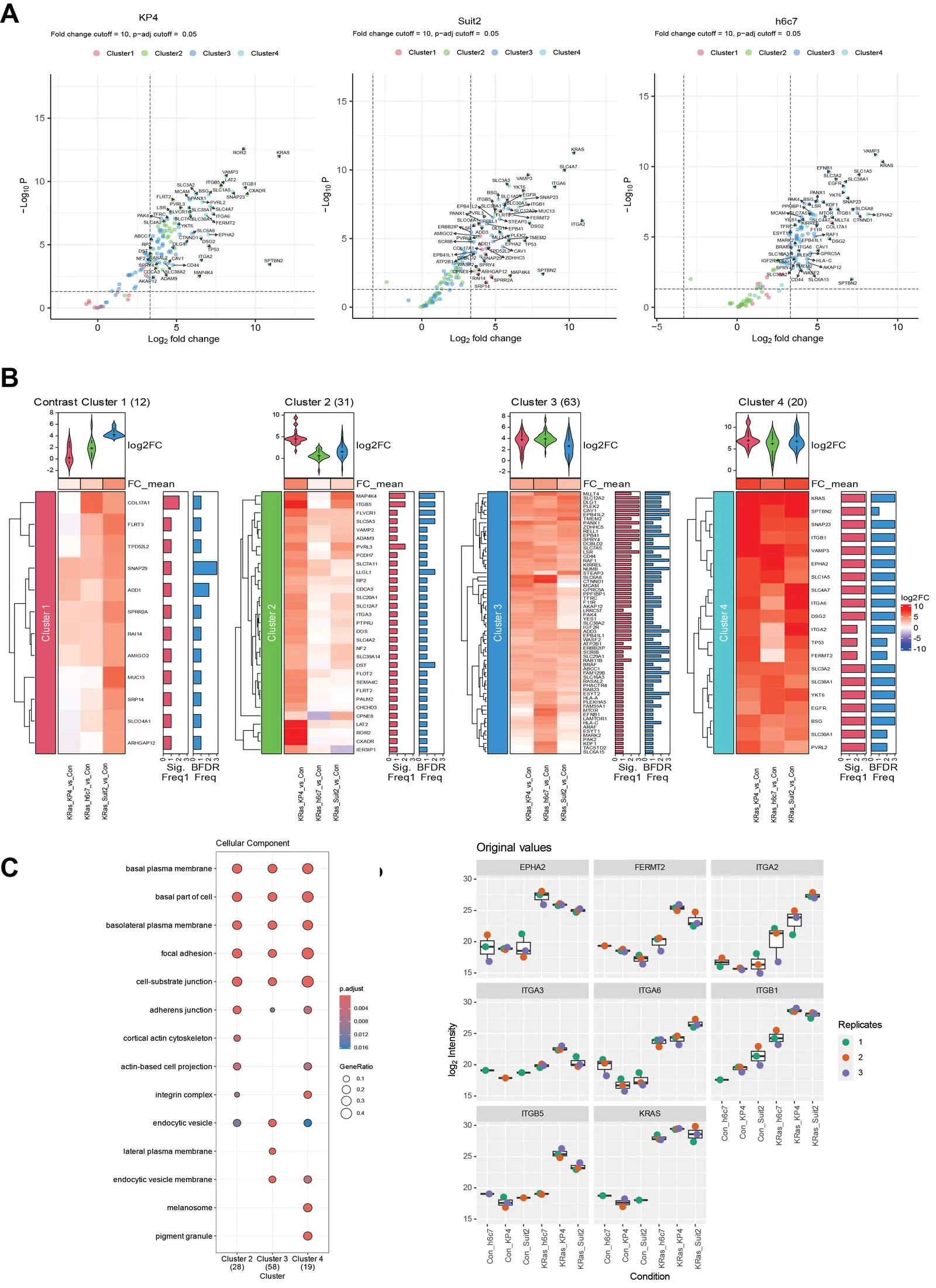
**Mass spectrometric analysis of KRas^G12V^-associating proteins in human pancreatic cancer cell lines**. Streptavidin-binding proteins from biotinylated samples of KP4, Suit-2, and h6c7 cells were subjected to LC-MS/MS analysis and the data were analyzed using MaxQuant. *A*, volcano plots of significantly enriched proteins. *B*, K-means clustering of significantly enriched proteins with a BFDR threshold of ≤0.05. *C*, functional enrichment analysis of protein lists obtained from BioID and LC-MS/MS analysis. Protein lists were imported into “R” and the clusterProfiler package was used to perform gene ontology enrichment analysis. The chart represents the cellular components category of the enriched proteins. The dot size represents the gene ratio for the associated GO term, and the color represents the adjusted *p*-value. Cluster 1 was omitted as it had too few components to assess. *D*, box plots of proteins of interest showing the mean intensity values from the KRas- and BirA∗-expressing samples of each cell line. BFDR, Bayesian false discovery rate; BioID, proximity biotinylation; GO, Gene Ontology.

Following selection using SAINTexpress analysis with a Bayesian false discovery rate (BFDR) of ≤0.05 and a fold-change (FC) between bait and control of >10, 126 proteins common to all cell lines were identified (Fig. 1, *A* and *B*). Unbiased K-means clustering of these proteins revealed four groups with distinct association patterns containing 12, 31, 63 and 20 proteins (Fig. 1, *A* and *B*). Clusters 1 and 2 were primarily driven by cell-specific associations, whereas Clusters 3 and 4 were more universal. Cluster 1 primarily comprised plasma membrane-associated proteins, while the proteins in the other clusters were mainly known KRas interactors (RAF1, BRAF, ARAF, AFDN, SNAP23, and VAMP2), RTK signaling proteins (EPHA2, EGFR, and associated interactors), adhesion receptors, regulators and adapters (integrins ITGA2, ITGA3, ITGA6, ITGB1 and ITGB5, FERMT2, CTNND1, CD44, YES1, and PTPRJ), trafficking regulators (Rab family members, CAV1, and FLOT2), and transporters (SLC38A2, SLC12A2, SLC7A5, and SLC4A7). The significant hits were then analyzed with ClusterProfiler to assign an objective list of ontological functions (Fig. 1*C* and Fig. S1*F*). Consistent with the localization of the KRas bait, the most abundant fraction of proteins indicated the cellular compartments enriched as different parts of the plasma membrane, followed by focal adhesions, cell-substrate junctions and adherens junctions (Fig. 1*C*). Biological processes included cell–matrix interactions, integrin signaling, cadherin binding, and transport of proteins and nutrients across membrane (Fig. S1*F*). Given this overall ontological analysis and the reported significance of adhesion receptors and RTKs in PDAC progression, it was notable that the bait-expressing samples contained a range of integrin subunits and RTKs. As the ion intensity values for these proteins, together with KRas and kindlin-2, were distinctly higher than other associating proteins when compared to BirA∗-expressing controls (Fig. 1*D*), this is suggestive of a potential functional connection between KRas and adhesion/RTK signaling (Fig. S1*G* illustrates the list of common and distinct significant hits obtained after the comparison of three cell lines included in the analysis).

### Definition of an oncogenic KRas interactome in PDAC organoids

**Figure 2:**
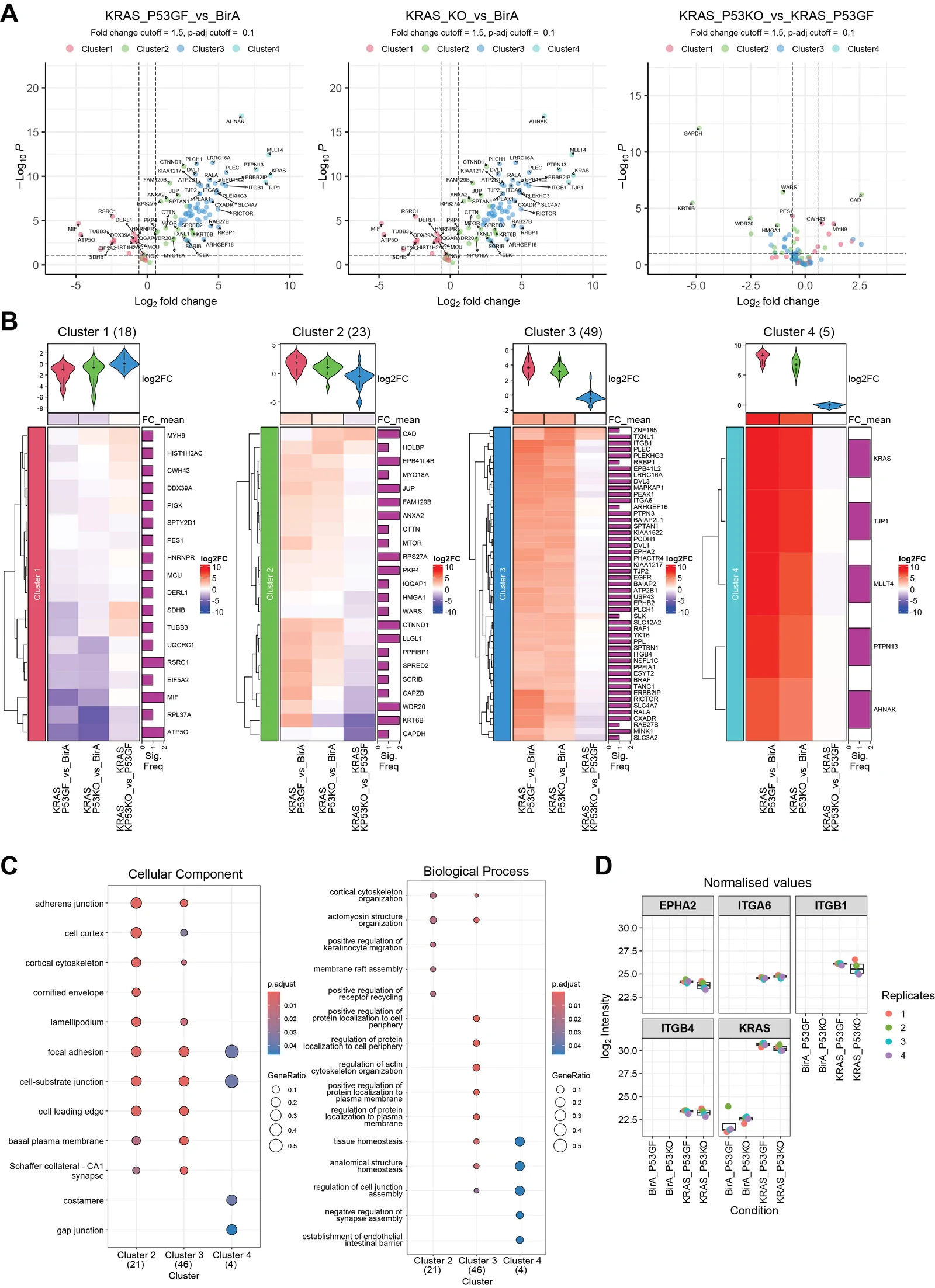
**Mass spectrometric analysis of KRas^G12V^-associating proteins in PDAC organoids**. Streptavidin-binding proteins from biotinylated samples of PDAC organoid models were subjected to LC-MS/MS analysis and raw data were analyzed using MaxQuant. *A*, volcano plots of significantly expressed differential proteins. *B*, K-means clustering of significantly enriched proteins with a BFDR threshold of ≤0.05. *C*, protein lists for KO and R172H organoids were imported into “R” and clusterProfiler package was used to perform gene ontology enrichment analysis. The charts represent the cellular components and biological processes of significantly enriched proteins. The dot size represents the gene ratio for associated GO terms, and the color represents the adjusted *p*-value. *D*, box plots of proteins of interest showing the mean intensity values from the KRas^G12D^-expressing organoid models. PDAC, pancreatic ductal adenocarcinoma; BFDR, Bayesian false discovery rate; GO, Gene Ontology.

To complement the data obtained with PDAC cell lines, the BioID approach was repeated using a murine PDAC organoid model. For this purpose, KRas^G12D^-expressing organoids were generated either with a gain-of-function mutation in p53 (R172H) (hereafter; p53 GF) or a p53 knockout (hereafter; p53KO) to mimic human tumors. KRas^G12D^ was conditionally expressed using a doxycycline-inducible system, and then organoids were incubated with biotin for 24 h and biotinylated proteins affinity-captured, trypsinized, and subjected to LC-MS/MS analysis. The raw data were searched against the UniProt database in MaxQuant followed by stringent statistical analysis using SAINTexpress and DEP software in “R” as earlier. A BFDR score of ≤ 0.05 and a FC ratio of 1.5 was applied to filter significant hits. The data were processed for pairwise comparisons using ion intensity values and PCA indicated a distinct separation between the sample types (bait *versus* control) and a strong Pearson correlation indicating sufficient reproducibility of the data (Fig. S2, *A* and *B*). The filtered ion intensities were adjusted to normalize the replicates without impacting the overall intensities of the data (Fig. S2*C*). A total of 95 proteins were found to differ significantly between samples after pairwise comparisons and were used in further analyses (Fig. 2).

K-means clustering revealed four groups with distinct association patterns. The comparison identified many proteins enriched in the p53 GF group and the p53KO group compared to the respective BirA∗ control group. Most of the proteins were common to both groups (Fig. 2*A*). The hierarchical classification of the four identified clusters identified 5, 49, 23, and 4 proteins (Fig. 2*B*). A small number of proteins identified in PDAC cell lines (Fig. 1) were also identified in PDAC organoids, including canonical KRas interactors (Raf1, b-Raf, a-Raf, and afadin (MLLT4), EGFR, integrins (α6, β1, and β4), and ephrin receptors (EphA2 and EphB2) (Figs. 2, *A* and *B* and Fig. S2*E*). Functional enrichment analysis was performed on the significant hits using ClusterProfiler. The gene ontology terms most enriched were focal adhesions, cell-substrate junctions, actin cytoskeleton, and adherens junctions in the cellular compartments category, whereas the biological processes associated with these terms were localization of the proteins to cell periphery/membrane, positive regulation of cell-cell adhesion, and metabolic processes associated with pyrimidine synthesis (Fig. 2*C*). Similarly, the molecular functions associated with the most enriched GO terms were cadherin binding, actin binding, and cell-cell adhesion regulation (Fig.S 2*D*). The ion intensity values for the selected protein of interest, *i.e.* EphA2 and ITGB1 (along with other integrin subunits), were solely identified in KRas-expressing samples as compared to respective controls (Fig. 2*D*). In summary, a highly similar set of KRas-associated proteins, primarily linked to cell adhesion and RTK signaling, as well as canonical Ras interactors, were identified using both cell lines and organoids.

### Validation of candidate association with KRas in PDAC cell lines

To test the hypothesis that KRas associates with integrins and RTKs, two candidates were selected for follow-up: EphA2, a RTK that regulates multiple signaling cascades in a ligand-dependent and -independent manner, and integrin β1. Both receptors have been previously implicated in PDAC progression and the desmoplastic response of PDAC (57, 58, 59).

**Figure 3:**
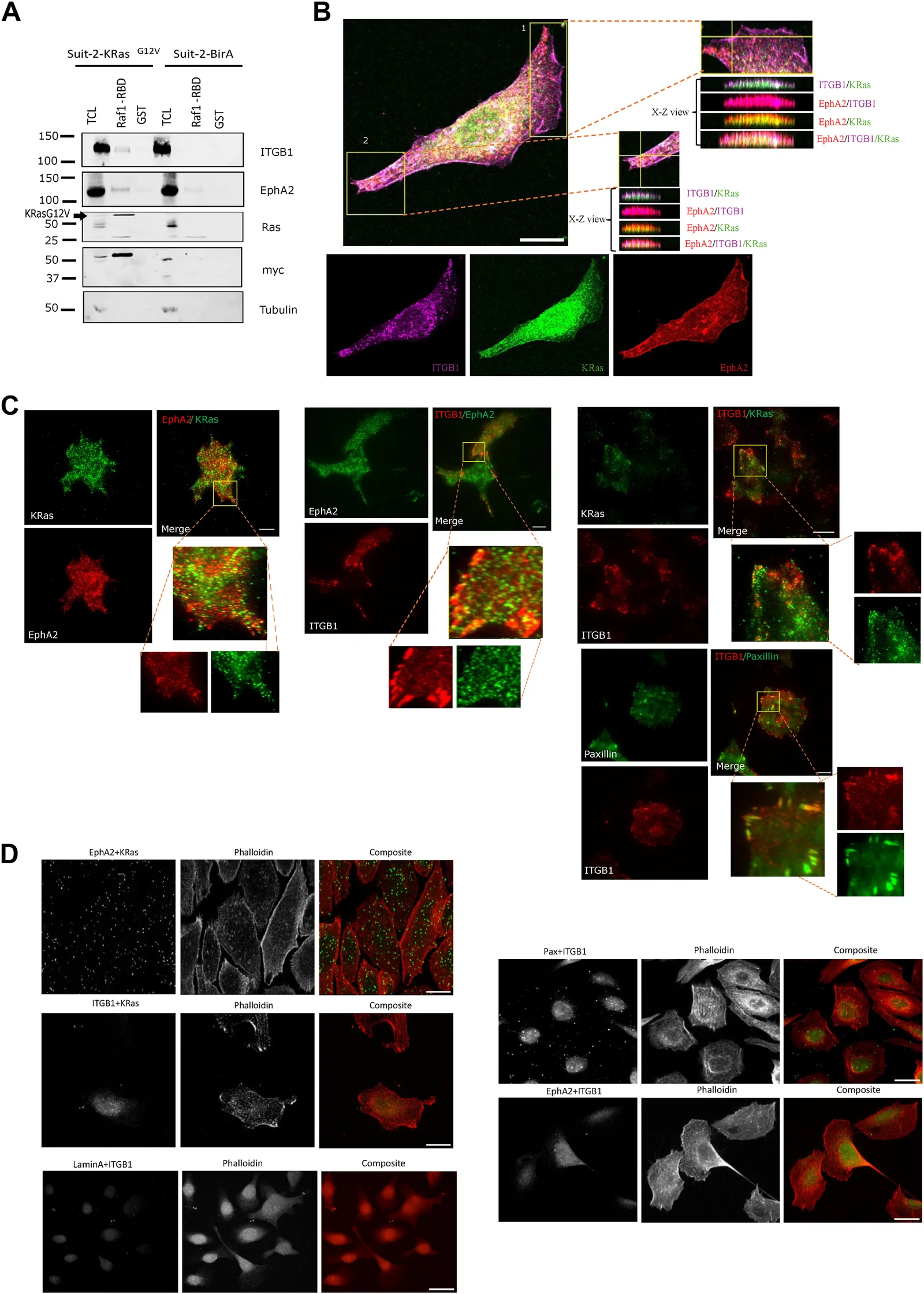
**Localization of KRas, EphA2, and integrin β1 in PDAC cell lines**. *A*, Ras activation assay of Suit-2 cells expressing BirA∗ and BirA∗-KRas analyzed by Western blotting. *B*, confocal immunofluorescence analysis of Suit-2 cells stained with antibodies directed against KRas (*green*), EphA2 (*red*), and integrin β1 (*magenta*). Regions of interest (ROI) were selected to show greater detail, and an orthogonal view of the image was created to show their interaction in x-z views. The scale bar represents 10 μM. *C*, TIRF analysis of KP4 cells for KRas (*green*), EphA2 (*red* or *green*), integrin β1 (*red*), and paxillin (*green*). ROI were selected to show greater detail. The scale bar represents 25 μM. *D*, proximity ligation assay of selected proteins in Suit-2 cells. PDAC, pancreatic ductal adenocarcinoma; TIRF, total internal reflection fluorescence.

To test if the candidates interact with active KRas, the Ras binding domain of Raf-1 (Raf1-RBD) was used to pull down Ras-associating proteins from Suit-2 cells (Fig. 3*A*). Endogenous Ras protein was detected in the total cell lysate (TCL) of both BirA∗-KRas- and BirA∗-expressing cells and pulled down by the Raf1-RBD-binding domain in both samples, indicating that active Ras was affinity enriched by its effector protein (Fig. 3*A*). In addition, a band at approximately 59 kDa in the TCL and eluted fractions of BirA∗-KRas-expressing cells was detected, suggesting the presence of transduced KRas^G12V^. The selected candidates (EphA2 and integrin β1) were detected in Raf1-RBD pull-down fractions in addition to the TCL fractions (Fig. 3*A*). While low levels of EphA2 were observed in the Raf1-RBD fraction of BirA∗-expressing cells, which could be mediated by endogenous G12D-mutated KRas, the expression of the detected protein was much higher in Raf1-RBD fractions of BirA∗-KRas^G12V^-expressing cells. Thus, both integrin β1 and EphA2 associate with active KRas.

**Figure 4:**
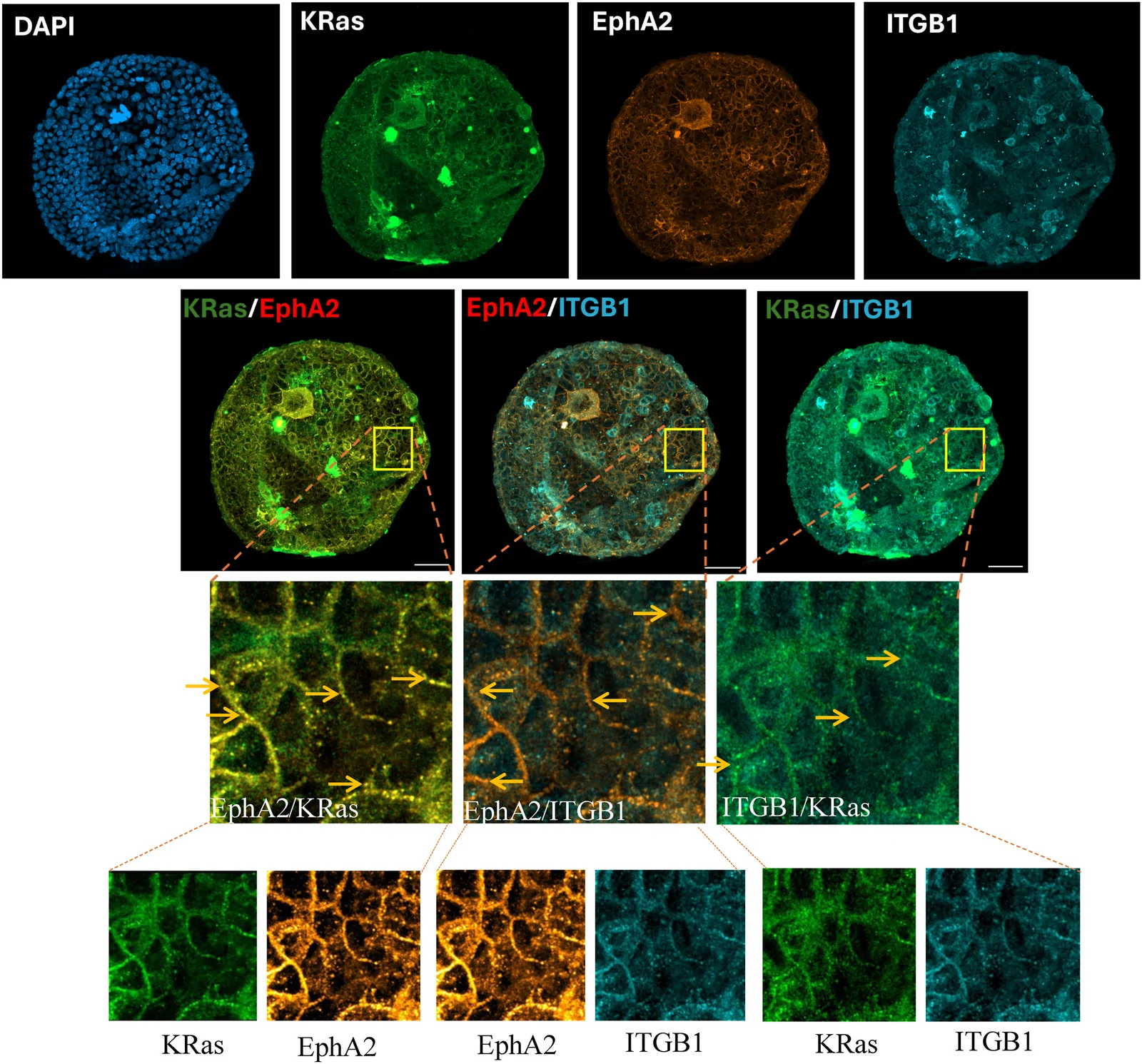
**Localization of KRas, EphA2, and integrin β1 in PDAC organoids**. Confocal immunofluorescence analysis for KRas (*green*), EphA2 (*red*), and integrin β1 (*cyan*). The merged images show regions of colocalization between KRas, EphA2, and integrin β1, alongside the corresponding individual fluorescence channels from two representative PDAC organoids. *Arrows* indicate the regions where colocalization is observed. Selected ROI are shown at higher magnification. The scale bar represents 50 μM. PDAC, pancreatic ductal adenocarcinoma.

Next, to test the potential colocalization of KRas with candidate proteins, confocal immunofluorescence imaging was performed. Suit-2 cells were seeded onto laminin (LN)-coated coverslips for 2 h prior to fixation. The cells were then stained for endogenous KRas, EphA2 and integrin β1 (Fig. 3, *B* and *C*). KRas localized to the vicinity of both EphA2 and integrin β1 in small puncta near the cell periphery (Fig. 3*B*). This localization was stronger between EphA2 and KRas than integrin β1. The colocalization at the cell-substrate interface was further probed by total internal reflection fluorescence (TIRF) microscopy (Fig. 3*C*). KP4 cells, which spread better than Suit-2, were seeded on LN-coated coverslips for 2 h before fixation and staining. Paxillin was used as a positive marker to identify integrin adhesion complexes. As before, all three proteins overlapped in a punctate appearance. To compare this colocalization between an oncogenic and WT KRas background, a human uterine sarcoma cell line (U2OS) was examined (Fig. S3). U2OS cells were seeded onto fibronectin (FN)-coated coverslips for 2 h before fixation and staining. The same punctate colocalization between KRas, EphA2, and integrin β1 was observed, albeit with a lower degree of overlap compared to the PDAC cell lines. The interaction between EphA2, KRas, and integrin β1 was also visualized in Suit-2 cells by proximity ligation (Fig. 3*D*). Although it is difficult to make quantitative comparisons between different pairs of molecules because of antibody variation, KRas associated qualitatively more strongly with EphA2 than it did with integrin β1, or EphA2 did with integrin β1, which might suggest the presence of two different complexes. Colocalization was, however, present all over the ventral plasma membrane. As a positive control, integrin β1 interacted with paxillin, and the intensity of the spots as well as their density overlapped with the spots produced by the EphA2–KRas interaction. Lamin-A is a nuclear envelope protein (60) that does not interact with membrane-localized active integrin β1 protein; thus, it was used as a negative control. Finally, confocal imaging was repeated in in KRas^G12D^ PDAC organoids (Fig. 4). As observed in PDAC cell lines, a high degree of colocalization was found between KRas and EphA2, and integrin β1 and EphA2 across the plasma membrane, while KRas-integrin β1 colocalization was positive, but much lower. Taken together, these results demonstrate colocalization between KRas, EphA2 and integrin β1 in adhesion complexes and elsewhere on the ventral plasma membrane that may serve as hubs for activation and regulation of downstream signaling.

### EphA2 controls the levels of KRas and integrin β1

**Figure 5:**
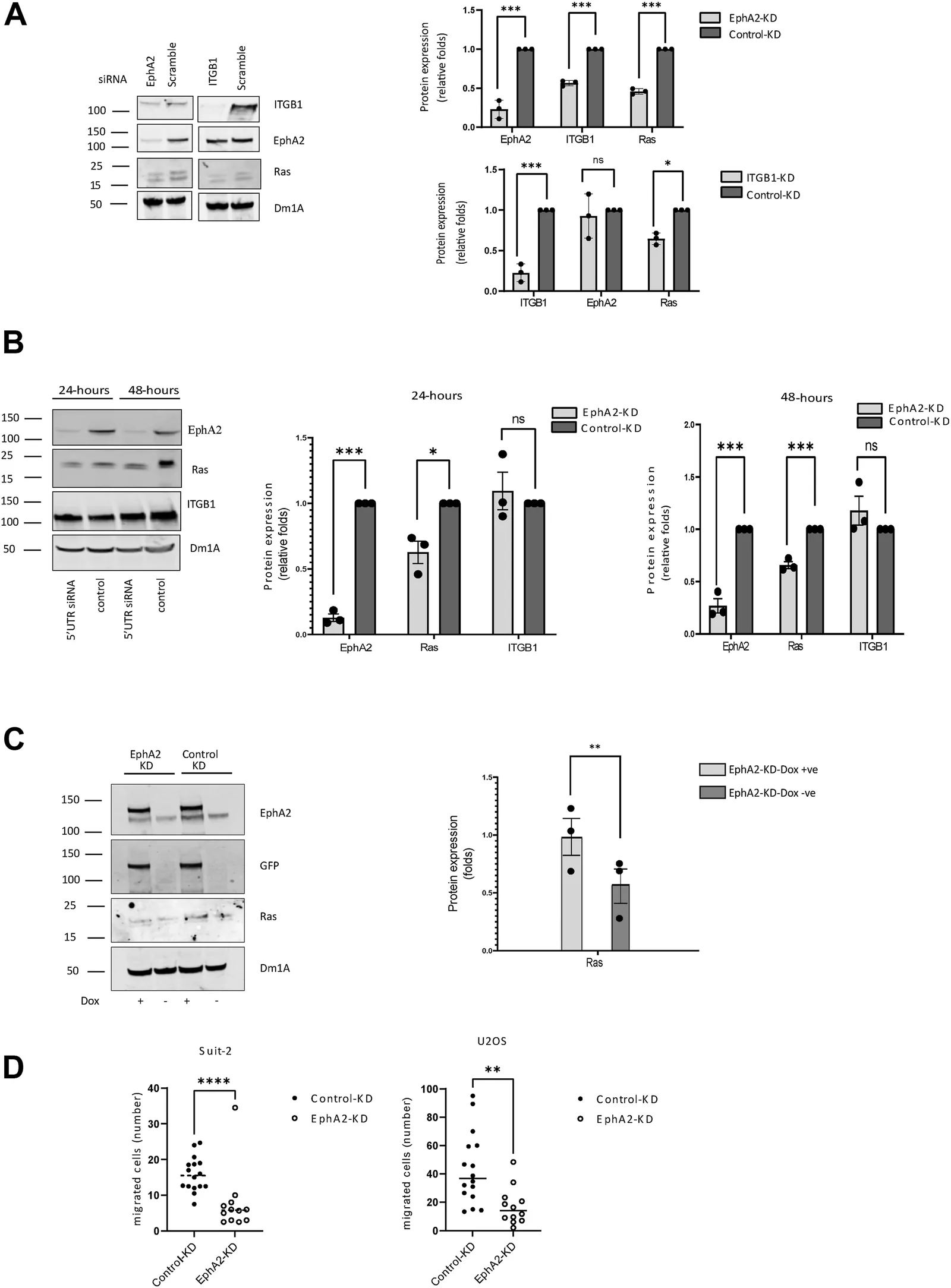
**EphA2 regulates KRas and integrin β1 expression**. *A*, Suit-2 cells were treated with respective siRNAs, lysates were prepared at 48 h posttransfection and subjected to Western blotting. Protein levels were normalized to tubulin expression. n = 3. Error bars represent the standard error of the mean (SEM) and two-tailed *p*-values were obtained using the Student’s *t* test: *p*-value significance <0.05. Scramble, non-targeting control siRNA sequence. The indicated *p*-values; ns = nonsignificant; ∗ ≤0.01; ∗∗∗ ≤ 0.001. *B*, EphA2 was depleted in Suit-2 cells using double knockdown with 100 nM 5′UTR siRNA 24 h apart. n = 3. Dm1A, tubulin. Error bars represent the standard error of the mean (SEM). *p*-value; ns = nonsignificant, ∗ ≤0.05, ∗∗∗ ≤ 0.001 obtained using the Student’s *t* test. *C*, endogenous EphA2 was depleted using a 5′UTR siRNA, and an exogenous YFP-tagged EphA2 was expressed using doxycycline treatment (Dox + ve) for 24 h to assess the ability to rescue depletion by Western blotting, while control cells (Dox -ve) were untreated cells. The data were compared in an unpaired *t* test, ∗∗ *p*-value = 0.0032. n = 3. *D*, EphA2 was depleted in mutated or wild-type KRas-expressing cell lines (Suit-2 and U2OS), and cells were serum starved for 24 h before adding fresh medium. Cell migration was assessed using a Transwell/Boyden chamber assay. Cells were seeded in the upper chamber on a collagen plug in serum-free medium, and complete growth medium was used as a chemoattractant in the lower chamber. The base of the lower chamber was coated with fibronectin and laminin mixture. Migration was quantified 32 h after seeding. The data were compared in an unpaired *t* test, ∗∗∗∗ *p*-value<0.0001; ∗∗ *p*-value = 0.0031 ns, nonsignificant. n = 3. UTR, untranslated region; YFP, yellow fluorescent protein.

As the degree of EphA2-KRas and EphA2-integrin β1 colocalization was greater than KRas-integrin β1, this suggested a role for EphA2 in orchestrating the ternary association. To test whether this was also the case for protein expression, each protein was knocked down using siRNA in Suit-2 cells (Fig. 5*A*; Fig. S4*A*). EphA2 depletion resulted in a 78% reduction of EphA2 protein, compared to a non-targeting siRNA, and this also significantly reduced the abundance of Ras (55% reduction) and integrin β1 (44% reduction) (Fig. 5*A*). Integrin β1 depletion resulted in a 78% reduction in integrin β1 protein, a 35% depletion of Ras, but only an 8% reduction in EphA2 (Fig. 5*A*). Similarly, KRas depletion resulted in a 50% loss in the level of KRas protein, 52% reduction in EphA2 levels and no change in integrin β1 (Fig. S4*A*). Thus, EphA2 may stabilize the level of KRas and integrin β1 through potential connecting proteins. This experiment was repeated with Suit-2 cells using double knockdown of EphA2 with a 5′UTR siRNA (Fig. 5*B*). This treatment resulted in a reduction of Ras expression to 38% at 24 h and 35% at 48 h. The expression of integrin β1 was unchanged (Fig. 5*B*). YFP-EphA2, but not control alone, was able to rescue the EphA2 KD effect and restore the expression of Ras (Fig. 5*C*). Furthermore, the rate of cell migration in a Boyden chamber assay was reduced in both Suit-2 and U2OS following EphA2 depletion (Fig. 5*D*). Thus, these data indicate the presence of EphA2, integrin β1 and KRas in proximity to each other, with EphA2 possibly regulating KRas and integrin β1 signaling to influence cell migration.

### Shc1 links EphA2, KRas, and integrin β1

**Figure 6:**
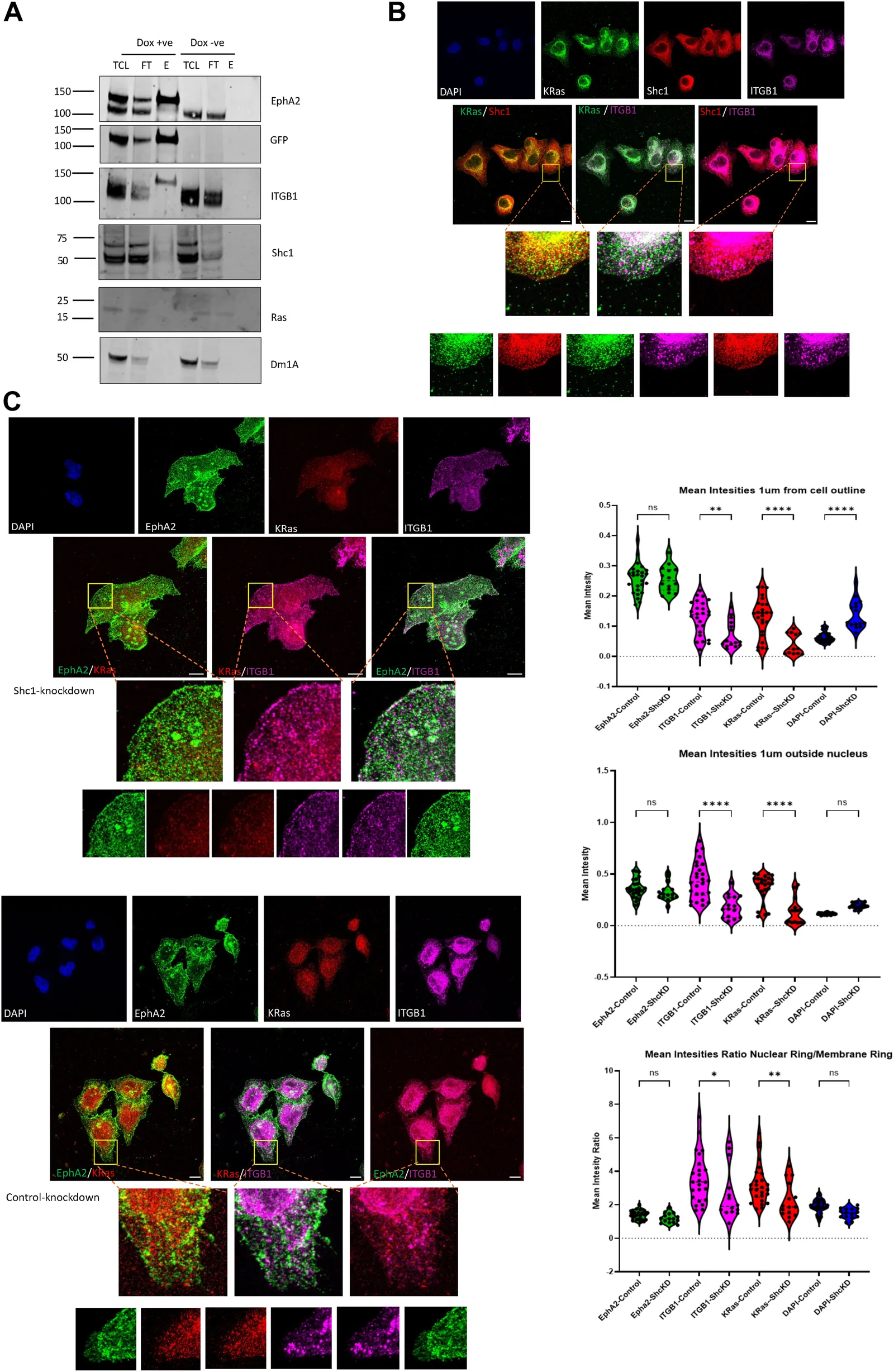
**Shc1 links EphA2 to KRas and integrin β1**. *A*, Suit-2 cells were treated with doxycycline (Dox + ve) to express YFP-EphA2 or control cells without doxycycline (Dox -ve) and were subjected to a GFP pulldown. The samples were Western blotted with indicated antibodies. Anti-Dm1A antibody was used to normalize to tubulin expression. n = 5. *B*, Suit-2 cells were seeded onto laminin-coated coverslips and serum-starved before staining and fixation. Staining was performed using antibodies directed against KRas, Shc1, and integrin β1, visualized with Alexa Fluor 488 (*green*), Alexa Fluor 594 (*red*), and Alexa Fluor 647 (*magenta*) conjugated antibodies, respectively. Images were captured in 3-D optical stacks with an LSM 880 confocal microscope (Airyscan mode). Staining and colocalization were visualized as an overlap of all channels or two channels together. ROIs were selected to observe the interaction in more detail. The scale bar represents 10 μM. *C*, Suit-2 cells were treated with Shc1 siRNA and seeded onto laminin-coated coverslips. Serum starvation was performed before staining and fixation of cells. Staining was performed using antibodies directed against EphA2, KRas, and integrin β1, visualized with Alexa Fluor 488 (*green*), Alexa Fluor 594 (*red*), and Alexa Fluor 647 (*magenta*) conjugated antibodies, respectively. Images were captured in 3-D optical stacks with an LSM 880 confocal microscope (Airyscan mode). Staining and colocalization between candidates were visualized as an overlap of all channels or two channels together. ROIs were selected to observe the interaction in more detail. Images are representative of staining seen across samples. The scale bar represents 10 μM. The graphs represent the mean intensity of the fluorophores measured across 1 μM edges of the cell and nuclear membrane to detect changes in the expression of proteins of interest following Shc1 knockdown. Mean intensity values were obtained in the image analysis using the CellProfiler pipeline. YFP, yellow fluorescent protein; LSM, laser scanning microscope.

As an initial step to delineate the mechanism by which EphA2 regulates the level of KRas and integrin β1, a candidate approach was employed to test the hypothesis that Shc1 coordinates receptor association. Shc1 is recruited by many RTKs (61, 62, 63), as well as integrins (64), and based on elegant phosphoproteomic approaches has been demonstrated to orchestrate temporal switches in the activation of RTK and integrin/cytoskeletal signaling (64, 65, 66, 67, 68). To test if Shc1 has an analogous role in PDAC cells, its ability to associate with EphA2, KRas and integrin β1 was tested by immunoprecipitation and Western blotting. Using a GFP trap, YFP-tagged EphA2 was used to pull down interacting partners (Fig. 6*A*). Exogenous and endogenous EphA2 protein were pulled down together with integrin β1 and Shc1, but KRas was not detected (Fig. 6*A*).

Potential colocalization of EphA2, KRas, integrin β1, and Shc1 was examined by confocal immunofluorescence microscopy. Considerable overlap in punctate patches was observed between KRas, Shc1, and integrin β1 in Suit-2 cells (Fig. 6*B*). To test the role of Shc1 in EphA2, KRas, and integrin β1 association, Shc1 was depleted and colocalization quantified by immunofluorescence microscopy using a custom CellProfiler pipeline. Cells were automatically segmented, and signal intensity was measured within a 1 μm membrane-proximal region to capture cell edge-associated staining. Perinuclear intensity was also quantified to determine the enhanced signal observed in KRas- and integrin β1-expressing cells. The pipeline also calculated the nuclear-to-membrane intensity ratio. Shc1 depletion resulted in dissociation of the interaction between EphA2 and integrin β1, caused a loss of KRas expression within 1 μm of the cell edge, and led to decreased colocalization between EphA2 and KRas (Fig. 6*C*). These results suggest that Shc1 may play a role in linking EphA2 to integrin β1 and KRas signaling in PDAC cells.

## Discussion

The major findings from this study are: (a) KRas, in in 2D human and 3D organoid cultures, associates with a common set of proteins that have established roles in signal transduction, adhesion, cytoskeletal organization, metabolism, and protein trafficking; (b) KRas, EphA2, and integrin β1 receptors form a ternary association that is primarily orchestrated by EphA2 and connected by Shc1; and (c) depletion of EphA2 inhibits KRas-dependent migration.

The integrin subunit identified in this study with the highest statistical significance in both PDAC cell lines and PDAC organoids was integrin β1. Moreover, two of its pairing alpha subunits (α2 and α6), and one of its primary effectors (kindlin-2) were detected. Previous proximity proteomic analyses of oncogenic KRas isoforms have also reported integrins (40, 53, 56), as well as RTKs, as associating proteins but the nature of these associations and their potential functional relevance have not been explored (40, 53, 56). In view of the reported overlaps in KRas, EphA2, and integrin signaling pathways, and their links to PDAC progression (31, 69, 70, 71, 72, 73), this suggests that their association may be relevant for function.

Immunofluorescence analyses determined that all three proteins do indeed colocalize in cells. Punctate clusters were detected at the cell periphery, with highest colocalization found between KRas and EphA2, compared to integrin β1/EphA2 or KRas/integrin β1. Similarly, protein biochemical assays demonstrated coprecipitation of integrin β1 and EphA2 with active KRas. These results are suggestive of at least the transient presence of each candidate in the same complex. Consistent with this conclusion, the depletion of EphA2 reduced the expression of KRas and integrin β1, and the depletion of integrin β1 reduced the expression of KRas. This suggests that KRas association with either receptor stabilizes its presence at the plasma membrane and may imply a mechanism for coordinated turnover. A range of reports have already connected KRas, EphA2, and integrin β1 to PDAC progression. For example, the overexpression of EphA2 is associated with a poor prognosis in cancer, including PDAC, and context-dependent links to KRas signaling have been reported (70, 74, 75). EphA2 regulates two independent KRas signaling pathways for hepatocellular carcinoma proliferation, Akt and Jak/Stat3 (76), and regulates the growth and proliferation of KRas-dependent cell lines in a feedback loop mechanism by promoting the activation of the MAPK pathway (77). In normal cells, as well as in precancerous lesions, EphA2 has a protective role and mediates the extrusion of mutant KRas-containing cells from normal cells possessing WT KRas, a process that may also involve integrins (46, 78, 79). In the current study, EphA2 was not only able to regulate the expression of KRas but was also required for the migration of PDAC cells, while the overexpression of EphA2 was able to rescue the KRas levels following depletion of the endogenous protein. These findings suggest that EphA2 is an upstream positive regulator of KRas in PDAC. Future studies could explore the mechanisms whereby ternary signaling from the three candidates coordinates cell morphology and adhesiveness, and how EphA2 trafficking regulates KRas surface expression.

Once stimulated, EphA2 undergoes transphosphorylation in its kinase domain, which recruits adapter proteins to initiate signaling (64). One such adapter protein is Shc1, which recruits Grb2 and Sos, and thereby drives the MAPK and Akt pathways to control cellular growth and proliferation (67). Members of the ErbB family and EphA2 were among 40 proteins identified in a proteomic analysis of Shc1 interactors (67), and EphA2 has been identified in a range of screens as associating with Shc1 in lung adenocarcinoma and gastric cancer (62, 63, 67). Moreover, Shc1 is recruited in a FAK/integrin-dependent manner in response to changes in the cell microenvironment to activate the MAPK pathway (80). Here, EphA2 was not only able to coprecipitate with integrin β1 and Shc1, but the depletion of Shc1 was sufficient to disrupt the interaction between EphA2 and integrin β1. KRas was shown to colocalize with Shc1 at the plasma membrane, while depletion of Shc1 resulted in loss of membrane-localized KRas, and therefore colocalization between EphA2 and KRas. Given the growing view of Shc1 as a key switch in signaling pathways that link proliferation and adhesion/cytoskeleton (67, 81), the role of Shc1 in PDAC progression is worthy of further investigation. Collectively, these data provide new insight into how KRas signaling is integrated with RTK and integrin pathways in PDAC.

## Experimental procedures

### Chemicals

DMEM supplemented with GlutaMAX (with or without sodium pyruvate), laboratory-grade PBS, penicillin/streptomycin, trypsin-EDTA, and fetal bovine serum (FBS) were purchased from Thermo Fisher Scientific or Gibco. Lipofectamine 3000 and RNAiMax kits were purchased from Invitrogen Life Technologies (Thermo Fisher Scientific). Anti-GFP and Streptavidin beads were purchased from ChromoTek and 2BScientific, respectively. The HiFi DNA assembly cloning kit was purchased from NEBuilders. The KOD Hot-Start Master Mix-71842-3 was purchased from Merck Millipore. Primary and secondary antibodies used in this study are listed in Table S1.

### Cell line culture

The human PC cell lines used in this study were KP4, which expresses KRas^G12D^, and Suit-2, which expresses KRas^G12D^, CDKN2A^H83Y^ and TP53^R273H^. These cell lines were maintained in DMEM with GlutaMAX and sodium pyruvate with 1% (w/v) glutamine and 10% (v/v) FBS. The human uterine sarcoma cell line U2OS and human embryonic kidney (HEK) cell line were cultured in D5796 with GlutaMAX and sodium pyruvate and supplemented with 1% (w/v) glutamine and 10% (v/v) FBS. H6c7 cells (44), which are derived from normal human pancreatic ductal epithelium and contain WT KRas, were maintained in keratinocyte serum-free medium (Gibco). All cell lines were grown in vented flasks at 37 °C in a humidified incubator with 5% (v/v) CO_2_. Cells were grown to confluence and split in a 1:3 ratio for U2OS and H6c7, and 1:5 for KP4, Suit-2 and HEK cells. All experiments were performed on cells between passage numbers 5-25. All cell lines were validated using short tandem repeat profiling.

### Organoid culture

Murine organoid lines TJ1365 (Kras^G12D/+^, Trp53^KO^) and TJ4124 (Kras^G12D/+^, Trp53^R172H/+^) were kind gifts from Tyler Jacks (MIT). Organoid lines were cultured in hPOCM growth medium/Advanced DMEM/F12 (AdF; Gibco) supplemented with GlutaMax, 0.1 M Hepes (Thermo Fisher Scientific), HyClone (Cytiva), 1 mM N-acetyl cysteine (Sigma-Aldrich), 100 ng/ml Wnt3a (R&D Systems), 100 ng/ml human noggin (PeproTech), 100 ng/ml FGF-10 (PeproTech), 10 mM nicotinamide (Sigma-Aldrich), 0.5 μM A83–01 (BioTechne), 10% (v/v) RSpo1 conditioned medium, and 0.01 μM gastrin (Sigma-Aldrich). The medium was changed every 3 days to facilitate consistent organoid growth. For passaging, following removal of the growth medium, Matrigel domes (Corning) were depolymerized in ice-cold AdF supplemented with GlutaMax, 0.1 M Hepes, and HyClone. Organoids were mechanically dissociated into fragments using a 200 μl pipette tip and replated at a 1:3 split into new 20 μl Matrigel droplets. Frozen organoid stocks were established by mixing dissociated organoids with Recovery Cell Culture Freezing Medium (Gibco) followed by gentle cryopreservation. Organoids were thawed by rapidly warming cryotubes and following washing organoids twice in AdF base medium. Organoids were seeded in 20 μl droplets of Matrigel.

### Plasmid constructs

Human KRas^G12V^ cloned in frame 3′ of myc-BirA∗ plasmid pcDNA3.1 from Addgene (37500) was provided by Hema Adhikari (Duke University). BioID vector pCDH-TagBFP-myc-BirA∗-Bax was a gift from Andrew Gilmore (University of Manchester). To create stable cell lines by lentiviral transduction, the KRas^G12V^ gene was amplified from the pcDNA3.1-myc-BirA∗-KRas^G12V^ vector into frame 3′ of myc-BirA∗ in pCDH-E1F-tagBFP-T2A-myc-BirA∗ using the HIFI DNA assembly method following the manufacturer’s instructions, creating the vector pCDH-E1F-tagBFP-T2A-myc-BirA∗-KRas^G12V^. EphA2-YFP was from Addgene (108852).

### Transient transfection

The gene of interest was transfected into PC cell lines using Lipofectamine 3000. Cells were seeded to approximately 70 to 80% confluence into cell culture plates 24 h before transfection and incubated overnight at 37 °C in a humidified incubator. The culture medium was discarded, and cells briefly washed with PBS before adding fresh medium with transfection mixture (2.5 μg DNA and lipofectamine at a 1:1 ratio in 125 μl Opti-Mem (Thermo Fisher Scientific)). A total of 5 μl of lipofectamine in 12 μl Opti-Mem was used for a single well of a 6-well plate and the mixture incubated for 12 min at RT before adding to the cells. After 24 h, the transfection medium was changed to normal growth medium, and cells were used for downstream applications. This protocol was scaled for 24-well, 10-cm and 15-cm dishes as per the requirement of the experiment.

### Generation of stable cell lines

For stable cell line generation, HEK 293 cells in T-75 dishes were cotransfected with pCDH-TagBFP-myc-BirA∗ and packaging vectors (psPAX2 and pM2G) using polyethyleneimine (PEI) to produce lentiviruses. HEK 293 cells were seeded to 60% confluence in a T-75 dish 24 h before transfection. On the day of transfection, 250 μl of Opti-Mem mixture with 6 μg pCDH-TagBFP-myc-BirA∗ plasmid, 4.5 μg psPAX2 and 3 μg pM2G plasmid was mixed with 250 μl Opti-Mem, 44.4 μM PEI and 1.5 mM NaCl and incubated at RT for 15 min. The growth medium of HEK 293 cells was replaced with 5 ml Opti-Mem and the PEI/DNA mixture added to cells dropwise. The medium was replaced with 15 ml fresh growth medium after 6 h of incubation. After 3 days of transfection, the medium was centrifuged at 1400 rpm for 4 min to remove debris. The supernatant was then filter-sterilized using a 0.45 μm filter, added to a fresh T-75 flask and the target cells seeded. The viral medium was removed from the cells after 24 h of incubation and the growth medium replenished. The cells were passaged 48 h later, and TagBFP-expressing cells were selected using fluorescence-activated cell sorting. 1 x 10^7^ cells were suspended in 1 ml DMEM with 25 mM Hepes and sorted based on BFP fluorescence using an LSR Fortessa cell analyzer and BD FACS-Diva 8 software (BD Biosciences). Puromycin-based selection was performed following viral transduction of the EphA2-YFP expressing vector with TetR promoter for the generation of a doxycycline-induced stable cell line.

### Organoid transduction

To generate BioID-expressing organoid lines, a second-generation lentiviral system was used. A total of 293FT cells were plated in a 10 cm culture dish and cultured overnight in CCM (DMEM, 10% (v/v) FBS; Hyclone). The next day, 4 μg of STV6-FLAG or STV6-KRAS^G12D^ transfer plasmid, 2 μg of psPAX2 packaging plasmid (Addgene 12260), 1 μg pMD2.G envelope plasmid (Addgene 12259), and 21 μl PEI were combined in a 3:1 (v/v) ratio (PEI:plasmids), mixed in 1 ml of serum-free DMEM and incubated at RT for 30 min. The plasmid/PEI mixture was added directly to 293FT cells and incubated overnight. Virus-containing supernatant was collected and stored at 4 °C, replaced with fresh CCM, and cells were cultured for a further 24 h. Virus aliquots from both 24 h and 48 h collections were combined, passed through a 0.22 μm syringe filter, and concentrated using a Lenti-X concentrator (Takara) according to the manufacturer’s instructions. Concentrated virus was stored in 250 μl aliquots at −80 °C until required. Target organoid cells were dissociated to single cells with TrypLE (Gibco) and 25,000 cells were combined with 250 μl concentrated lentivirus and 1 μl Polybrene (1 mg/ml) and centrifuged at 600 rpm for 1 h at RT. Virus was removed and cells were washed with AdF media prior to being embedded in 20 μl Matrigel and cultured with hPOCM. 48 h posttransduction, 1 μg/μl puromycin (Thermo Fisher Scientific) was added to select resistant organoid lines for 7 days, with the medium being changed every 3 days. Cells were expanded and cryopreserved until required for subsequent experimentation.

### Cell lysis

Cells grown to 80% confluence were washed with ice-cold PBS three times and lysed in ice-cold Pierce IP lysis buffer (25 mM Tris-HCl, pH 7.4, 150 mM NaCl, 1% (w/v) NP-40, 1 mM EDTA, and 5% (w/v) glycerol) supplemented with complete EDTA-free protease inhibitor cocktail (Sigma-Aldrich). The lysate was collected with a rigorous scraping of cells from culture flasks and kept on ice for 25 min. Lysates were centrifuged at 16000 g for 15 min at 4 °C and the supernatant was retained for analysis or frozen at −20 °C for further applications.

### SDS-PAGE and immunoblotting

Samples were diluted with SDS-PAGE loading buffer and loaded into 4 to 12% (w/v) NuPAGE Novex Bis-Tris gels and separated by SDS-PAGE using NuPAGE MES SDS running buffer at 150 V for approximately 60 min. Proteins were then transferred onto Amersham Protran 0.45 μm nitrocellulose membrane or Whatman Westran PVDF 0.2 μm membrane (Sigma-Aldrich) at 25 V for 12 min using a semidry transfer method in turbo buffer (1.5 M Tris-HCl, pH7.4, 0.125% (w/v) SDS, 1.5 M glycine, and 20% (v/v) ethanol). Membranes were then blocked with casein blocking buffer (Thermo Fisher Scientific) in PBS for an hour at RT and incubated with primary antibodies in casein blocking buffer in Tris-buffered saline-Tween 20 (TBS-T; 10 mM Tris-HCl, pH 8.0, 10 mM NaCl, and 0.05% (v/v) Tween 20) for an hour at RT or overnight at 4 °C. Membranes were washed 3 times with TBS-T for 5 min each and incubated with fluorophore-conjugated secondary antibodies in casein-blocking buffer in TBS-T for 30 min in the dark at RT. Membranes were washed 3 times with TBS-T and images were acquired using the Odyssey infrared scanning system (LI-COR Biosciences). Blots were further processed for the quantification of proteins using ImageJ software.

### Coomassie staining

Samples were loaded into 4 to 12% (w/v) NuPAGE Novex Bis-Tris gels and separated by SDS-PAGE using NuPAGE MES SDS running buffer at 150 V for 60 min. Gels were then incubated with Coomassie blue stain (Expedeon InstantBlue protein stain) at RT with constant shaking for 15 min. Gels were then destained with water overnight at 4 °C to reduce background stain and scanned with an Odyssey infrared scanning system (LI-COR Biosciences).

### Ligand coating

Where indicated, glass coverslips, glass-bottom dishes and 10 cm dishes were coated with 5 μg/ml LN or 10 μg/ml FN in PBS without calcium and magnesium (PBS-). The plates were incubated at 37 °C for 1 h for LN coating and at 4 °C overnight or 1 h at RT for FN coating. The coated coverslips were washed 3 times with PBS before seeding cells, followed by three washes with PBS to remove excess or unbound ligands. For BioID on different rigidities, 1 kPa, 8 kPa, and 25 kPa plates (Matrigen) or plastic dishes were coated with 1% (w/v) MaxGel ECM to increase cell attachment. The dishes were incubated for approximately 3 h at 37 °C in a humidified 5% (v/v) CO_2_ incubator and the excessive solution was aspirated. Plates were air-dried before seeding the cells.

### Widefield microscopy

To visualize the expression and localization of the KRas^G12V^ gene in PC cell lines, cells were seeded sparsely onto coverslips in 24-well plates and transfected with either pcDNA3.1-myc-BirA∗-KRas^G12V^ or pCDH-myc-BirA∗-KRas^G12V^ plasmids and their corresponding empty vectors as control. Transfection was performed as described earlier. 48 h after transfection, cells on coverslips were washed 3 times with PBS- for 5 min each and fixed with 4% (w/v) paraformaldehyde for 30 min at RT. The fixative was removed with PBS washes and cells were permeabilized with 0.2% (v/v) Triton X-100 in PBS for 30 min at RT. Coverslips were washed three times with PBS- and cells were incubated with 1:300 dilution of primary α-myc antibody (CST 9B11) in antibody dilution buffer (0.1% (w/v) bovine serum albumin and 0.2% (w/v) Triton X-100 in PBS) for 1 h at RT. Cells were washed three times for 5 min with PBS and incubated with a 1:500 dilution of Alexa Fluor 488-conjugated secondary antibody (Life Technologies) for 30 min at RT. Coverslips were washed 3 times for 5 min with PBS before air drying. Coverslips were directly mounted on glass slides with Prolong Diamond anti-fade mountant with DAPI (Thermo Fisher Scientific). Cells were visualized and images were taken on a Zeiss Axioimager.D2 upright microscope using 63X Plan-Apochromat (Oil, DIC) objective and captured using a Coolsnap HQ2 camera (Photometrics) through Micromanager Software v1.4.23. Specific bandpass filter sets for DAPI and FITC were used to prevent bleed-through.

### TIRF

For TIRF imaging, cells were allowed to attach to coverslips and serum starved for 2 h before fixation and staining as described earlier. Images were captured on a Leica Infinity TIRF microscope using a 100x/1.47 HC PL Apo Corr TIRF oil objective with 488 nm and 561 nm diode. A TIRF laser with 20% laser power, a penetration depth of 45 nm and an ORCA Flash V4 CMOS camera (Hamamatsu) with 200 ms exposure time and camera gain of 1 were used. The critical angle and horizontal approach angle for the TIRF beam were set to 90° to adjust the penetration depth and to correct for shadowing artefacts. Both epifluorescence and TIRF images were captured in a single field using the same parameters such as plane and exposure settings.

### Confocal microscopy

Confocal images of PDAC cell lines were captured using an Airyscan confocal microscope and selected regions were analyzed in an X-Z view. The cells were serum-starved for 2 h after sparsely seeding on ligand-coated coverslips overnight. The adherent cells were fixed and stained as described earlier. Images were acquired on an LSM 880 Airy upright microscope using an EC Plan-Neofluar 40x/1.30 Oil DIC M27 objective and 1.5x confocal zoom. The images were captured with the pinhole settings of 1 Airy unit and a scan speed of 1000 Hz (unidirectional). Images were acquired using hybrid detectors and the mirror settings were used as follows: DAPI (420–488 nm), FITC (495–550 nm), Texas Red (602–665 nm) and Cy5 (640–690 nm). White light laser lines of 488 nm (8.5%), 594 nm (9%), and 647 nm (6%) were used. The optimal number of z-stacks was selected using confocal software for 3D optical stack imaging. The images were reconstituted using the in-built Airyscan processing option of the confocal software and a region of interest was selected to view the colocalization between selected candidates in X-Z view.

PDAC organoids were harvested, fixed, cleared, and stained as previously reported (42). The images were acquired using a CD7 confocal microscope in Airyscan mode using the laser settings described earlier for cells and processed in Zeiss software as described before. Images were processed and analyzed using Image J/FIJI software for Widefield/TIRF images and ZEN black software v.2.3 SP1 for Airyscan confocal images.

### BioID

For BioID in PDAC cell lines, cells (stably expressing BirA∗ or KRas^G12V^-BirA∗) were seeded in 10 cm dishes (2 dishes per condition) and incubated at 37 °C in a humidified incubator overnight. The next day, the medium was discarded, and cells were washed with PBS. The cells were replenished with fresh medium containing 50 μM biotin (Sigma-Aldrich). After 24 h, cells were washed three times with PBS and lysed with 400 μl 50 mM Tris-HCl, pH7.4, 250 mM NaCl, 0.1% (w/v) SDS, 0.5 mM DTT, and cOmplete Protease Inhibitor Cocktail at RT. The lysate was transferred to a 15 ml tube and passed through a 19/21G needle four times. 2% (w/v) Triton-X100 was then added to the lysate and further passed through a 27G needle four times before adding 50 mM Tris-HCl, pH7.4. The lysates were centrifuged at 16000 g for 10 min and the supernatant was collected. A total of 50 μl supernatant per condition was saved as TCL to which 50 μl 2 x SDS reducing buffer was added and stored at −20 °C before further analysis. The remaining lysate was incubated with 100 μl prewashed and equilibrated MagReSyn Streptavidin Beads (ReSyn Biosciences) at 4 °C overnight with rotation. Beads were collected the next day, 50 μl of flow through was retained and 50 μl 2 x SDS sample buffer was added before storage at −20 °C for further analysis. Beads were washed twice with 2% (w/v) SDS for 8 min at RT with rotation. The flow-through was discarded and beads were washed with 0.1% (w/v) deoxycholate, 1% (w/v) Triton X-100, 500 mM NaCl, 1 mM EDTA, 50 mM Hepes, pH 7.5, and 0.5% (w/v) NP-40, 0.1% (w/v) deoxycholate, 1 mM EDTA, and 10 mM Tris-HCl, pH 7.4 for 8 min each at RT with rotation, and the flow-through was discarded. Proteins were eluted in 100 μl of 5 x SDS buffer at 85 °C for 10 min (2 rounds of 5 min each with 70 μl and 30 μl 5x SDS buffer, respectively) with constant shaking for in-gel tryptic digestion or eluted in 5% (w/v) SDS, 100 μM biotin, and 50 mM TEAB, pH 7.5 for S-trap protein digestion. The same protocol was adapted for BioID performed with KP4 cells growing on Perisoft collagen-coated dishes (1 kPa, 8 kPa, and 25 kPa) or plastic.

For BioID in PDAC organoids, BioID-expressing organoids were embedded in growth factor-reduced phenol red- and LDEV-free Matrigel (Corning) at a ratio of 20,000 cells per 20 μl Matrigel dome with 20 domes being used per biological replicate. On day 3 of culture, the medium was exchanged for hPOCM containing 1 μg/ml doxycycline and 50 μM biotin. On day 4, the medium was aspirated, and organoids were harvested with Cell Recovery Solution (Corning) containing protease inhibitor (Roche) and phosphatase inhibitors II/III (Sigma-Aldrich) for 20 to 30 min on ice. Organoids were pelleted at 300 g for 5 min and washed 3 times with PBS before freezing at −80C. Organoids were lysed in radioimmunoprecipitation assay lysis buffer (50 mM Tris-HCl, pH 7.6, 150 mM NaCl, 1 mM EGTA, 1.5 mM MgCl_2_, 1% (w/v) Triton X-100, 0.1% (w/v) SDS, and 0.5% (w/v) sodium deoxycholate) with Turbonuclease (Sigma-Aldrich) and protease inhibitor (Roche) and incubated for 20 to 30 min with end-over-end rotation at 4 °C. Insoluble material was removed by centrifugation at 16,000 g for 20 min. Lysate was incubated with 100 μl Streptavidin MagResyn beads (Resyn Biosciences) overnight at 4 °C with end-over-end rotation. Beads were washed with 25 mM Tris-HCl, pH 7.6, 2% SDS, 3 times with high salt radioimmunoprecipitation assay buffer (50 mM Tris-HCl, pH 7.6, 500 mM NaCl, 1 mM EGTA, 1.5 mM MgCl_2_, 1% (w/v) Triton X-100, and 0.1% (w/v) SDS), 3 times with 25 mM Tris-HCl, pH 7.6, 150 mM NaCl, 1 mM EDTA, 0.1% (w/v) NP-40, and 3 times with 50 mM TEAB/10% (v/v) acetonitrile. Streptavidin beads were pelleted and bound proteins were digested overnight at 37 °C in 50 mM TEAB, 10% (v/v) acetonitrile using 100 ng sequencing-grade trypsin (Promega) in a thermomixer. Following digestion, the supernatant was acidified to 0.1% (v/v) TFA and dried at 60 °C for 1 h. Peptides were resuspended in 0.1% (v/v) TFA, desalted using tC18 Spin Tips (Pierce) according to the manufacturer’s instructions, eluted in 50% (v/v) TFA, 10% (v/v) acetonitrile in LC-grade water and, following drying at 60 °C for 1 h, peptides were resuspended in 20 μl 0.05% (v/v) TFA for LC-MS/MS. Next, peptides were trapped on a PepMap 300 (C18, 300 μm × 5 mm; Thermo Fisher Scientific) column and separated on an Easy spray RSLC C18 column (75 μm × 500 mm; Thermo Fisher Scientific) at 180 nl/min using the following gradient profile (minutes:%B); 8:2, 45:29, 46:45, 47:80, 49:80, 50:2, and 70:2. The buffers used were 0.1% (v/v) formic acid in LC-grade water and 100% acetonitrile. The eluent was directed into an Easy-Spray source (Thermo Fisher Scientific) with temperature set at 60 °C and a source voltage of 1.7 kV. Data were acquired on a QExactive HFX (Thermo Fisher Scientific) with precursor scan ranging from 375 to 1200 m/z at 120,000 resolution and automatic gain control target of 3e6. The isolation window was set to 1.5 m/z. dd-MS^2^ scans were conducted at 30,000 resolution and automatic gain control target of 1e5 and normalized collision energy set to 29%. Two biological replicates were performed with two injections used for analysis per sample.

### Ras activity assay

Confluent cell cultures were washed three times with PBS and lysed. A total of 100 μl cell lysate was stored as TCL and the rest of the lysate was equally divided into two fractions per condition. A Raf-1 active Ras pull-down assay was performed using an active Ras detection kit (Cell Signaling Technologies) using the manufacturer’s protocol. The fractions from two divided samples were incubated with glutathione resin with either GST alone or GST-Raf1-Ras binding domain fusion protein for 1 h at 4 °C. These samples were then washed with Pierce IP lysis buffer 3 times and bound fractions were eluted in 2 X SDS sample buffer with 1% (w/v) dithiothreitol.

### siRNA-based depletion

For loss of function studies, commercially available on-target plus smartpool siRNA sequences (Dharmacon, Horizon Discovery) directed against the gene of interest were transfected independently into PC cell lines using Lipofectamine RNAiMax reagent (Thermo Fisher Scientific). Cells were seeded to approximately 80% confluence into cell culture plates 24 h before transfection and incubated overnight at 37 °C in a humidified incubator. The medium was discarded, and cells were briefly washed with PBS before adding fresh medium with 20 nM siRNA final concentration in 100 μl Opti-Mem (Thermo Fisher Scientific) and 5 μl of RNAiMax reagent in 100 μl Opti-Mem for a single well of a 6-well plate. The mixture was incubated for 10 min at RT before addition to the cells. A commercially available non-targeting smartpool siRNA sequence (On-Target Plus non-targeting siRNA; Dharmacon) was used as a negative control at the same concentration as the target siRNA. After 24 h, the transfection medium was changed to normal growth medium. Table S2 lists the siRNA used in this study.

### Cell migration

Suit-2 cells were seeded overnight in serum-free medium before subsequent seeding into Transwell Boyden chambers. A collagen plug was formed in the wells before adding the cells, and the lower chamber was coated with a 1:1 mixture of LN and FN (5 μg/ml and 10 μg/ml, respectively). A total of 40,000 cells were seeded in 100 μl serum-free medium into the wells and growth medium containing 10% (v/v) FBS was used as a chemoattractant in the lower chamber. The cells were allowed to migrate for 32 h and fixed with 4% (v/v) PFA. The lower chamber was stained with Hoechst nuclear stain and phalloidin (Rhodamine Phalloidin Reagent). Imaging was performed with a CD7 Discoverer microscope, and the number of cells was calculated using ImageJ/FIJI software and plotted using GraphPad Prism 9.

### GFP pulldown

Cells were seeded in 35 mm dishes to 70% confluency, and YFP-tagged EphA2 or empty vector were transiently transfected, or doxycycline-induced expression of EphA2 with YFP tag was performed. The cells were lysed, a fraction of the TCL was saved as input, and the remaining fraction was incubated with preequilibrated GFP-Trap Magnetic Particles M-270 beads according to manufacturer’s instructions (ChromoTek). Following 1 h incubation at 4 °C with end-over-end rotation, the supernatant was discarded (50 μl was saved as flowthrough) and beads were washed with washing buffer provided with the kit. The bound proteins were eluted in 100 μl of 2x SDS sample buffer by boiling at 95 °C for 5 min and the fractions were subjected to SDS-PAGE and Western blotting to detect the proteins.

### In-gel tryptic digestion

Samples were loaded onto 4 to 12% NuPAGE Novex Bis-Tris gels and run in NuPAGE MES-SDS running buffer for 3 min at 200 V. Gels were then stained with Coomassie stain (Expedeon InstantBlue) for 15 min at RT with constant shaking. Gels were destained with water at 4 °C overnight to reduce background staining and bands were sectioned and transferred to clean 96 well-perforated plates. Gel pieces were destained by incubation with 100 μl 25% (v/v) acetonitrile, 12.5 mM NH_4_HCO_3_ twice for 30 min at RT. Proteins were then dehydrated by incubating the gel pieces with 50 μl acetonitrile for 5 min at RT. Gel pieces were dried by vacuum centrifugation for 25 min and reduced by incubation with 50 μl 10 mM DTT for 1 h at 56 °C. Proteins were then alkylated by incubating gel pieces with 1 mM iodoacetamide for 45 min at RT in the dark. The proteins were further dehydrated by incubating the gel pieces with acetonitrile for 5 min at RT. Acetonitrile was removed by centrifugation, and gel pieces were rehydrated with 25 mM NH_4_HCO_3_ for 5 min at RT. The gel pieces were dehydrated again by adding 50% (v/v) acetonitrile and dried by vacuum centrifugation for 30 min at RT. Samples were digested with trypsin initially by incubating for 45 min at 4 °C, followed by overnight incubation at 37 °C. Peptides were collected by centrifugation and extracted by incubating the gel pieces with 50 μl 99.8% (v/v) acetonitrile in 0.2% (v/v) formic acid solution for 30 min at RT and then with 50 μl 50% (v/v) acetonitrile in 0.1% (v/v) formic acid for 30 min at RT. Peptides were collected by centrifugation and dried in a vacuum centrifuge. The dried samples were subjected to desalting before proceeding with MS.

### Suspension TRAPping filter (sTRAP)

The eluted samples from BioID experiments (with DTT) were heated at 95 °C for 10 min to allow reduction of proteins. Following the reduction of proteins, cysteines were alkylated by incubating the sample in the dark with 40 mM iodoacetamide. The samples were acidified with phosphoric acid to a final concentration of 1.2% (w/v) and transferred to sTRAP columns placed on top of 2 ml Eppendorf tubes to collect the flowthrough. Proteins were trapped on the protein trapping matrix of the sTRAP columns and washed thoroughly before digestion with sequencing grade trypsin at a final concentration of 0.5 μg/μl for 1 h at 47 °C on a Thermomixer. The peptides were eluted with 0.1% (v/v) formic acid and then with 50% (v/v) acetonitrile containing 0.1% (v/v) formic acid to recover hydrophobic peptides. The peptides were dried and subjected to desalting before proceeding to MS.

### Peptide desalting

A total of 5 mg of POROS R3 beads were added to each well of a 96-well plate with 0.2 μm PVDF membrane. 50% (v/v) acetonitrile was added to each well and the solution and beads were mixed well for 2 min at RT. The wash was removed by centrifugation at 200*g* for 1 minute and the wash was repeated. Samples were added to each well and mixed for 5 min at RT, centrifuged and the flow-through retained. 100 μl of 50% (v/v) acetonitrile was added and mixed twice for 2 min at RT. The solution was removed by centrifugation at 200*g* for 1 min and samples were dried by vacuum centrifugation. Peptides were resuspended in 11 μl 5% (v/v) acetonitrile in 0.1% (v/v) formic acid and stored at −20 °C.

### MS data analysis

Peptide samples were subjected to LC-M) using a Thermo Q-Exactive HF mass spectrometer (Thermo Fisher Scientific). Peptides were selected for fragmentation by data-dependent analysis. For MS-1 ion intensity-based data analysis, the data were searched using complete canonical, and isoform human proteome from the UniProt database (43). The raw data were analzsed against this proteome using MaxQuant (Version 1.6.17.0) (82). Most parameters were set as default, with an additional selected match between runs, label-free quantification, and lysine biotinylation modification were loaded. The grouped values were exported in a table format. A web-based tool “SAINT_DEP + Cluster” was utilized to further visualize the quality of the data and to filter out significantly enriched proteins from the dataset. To do so, the proteinGroups.txt file from MaxQuant and experimental design information were uploaded into “SAINT_DEP + Cluster”. To identify the certainty of interactions between bait and proximal prey, SAINTexpress (Version 3.6.3) (83) was used. The calculation was performed using the normalized protein label-free quantification intensities from MaxQuant as input. A 0.05 BFDR and a FC of 10 were applied as a threshold to identify high-confidence interactions. Briefly, SAINT_DEP + Cluster uses an unbiased approach where it uses the “DEP package” to filter out contaminants from the data, impute missing values based on the “Missing not At Random” method and normalize the data using the variance stabilizing transformation method (vsn) to screen the significant hits. To visualize the quality of the replicates, PCA and Pearson correlation plots were generated using the DEP suite. To identify the cluster of significant proteins, the protein list generated from SAINTexpress with a BFDR score of ≤0.05 was used to generate the heatmap of proteins with the “ComplexHeatmap package” and K-means clustering to group the proteins with similar FC values. To identify the functional roles of significant proteins, the “ClusterProfiler” package was applied to subject the significant proteins list to gene ontology enrichment analysis. To validate possible proteins of interest, enhanced volcano plots and box plots were generated to visualize the intensity of selected proteins between bait and control, and between all comparison conditions. Either experiments involving BioID bait or vector only were carried out with the same identification and quantification processes.

### Statistical analysis

Each experiment was performed in at least triplicate, and the results are expressed as mean ± standard error, or as stated. GraphPad Prism 9 was used to perform statistical tests where indicated. For the quantification of proteins, the bands were quantified by densitometry analysis in the ImageJ using an in-built plugin and protein expression was normalized to the tubulin expression quantified for that sample. For comparison between the control and treated groups, the normalized expression was compared between the groups to get an arbitrary unit for the expression of proteins. In the case of the GFP pull-down assay, as tubulin was not present in eluted fractions, the protein expression was simply compared in the treated *versus* control group. For the cell proliferation assay, the absorbance obtained for each triplicate (treated *versus* control groups independently) was averaged after background subtraction and the values obtained for treated groups and control groups were compared in a student *t* test and a two-tailed *p*-value was calculated in GraphPad Prism software. For the cell migration assay, cells were counted in images captured for each group separately and the number of cells was plotted and compared in an unpaired *t* test for comparison between treated and control at a single time point and a two-way ANOVA test was performed to analyze the variance between the treated and control groups from different time points.

## Data availability

All mass spectrometry data have been deposited to the ProteomeXchange Consortium *via* the PRIDE (84) partner repository with the dataset identifiers PXD069779 and 10.6019/PXD069779, PXD069991 and 10.6019/PXD069991 and PXD069885 and 10.6019/PXD069885 for cell lines, organoids, and cells growing on matrices with different rigidities, respectively.

## Supporting information

This article contains supporting information.

## Conflict of interest

The authors declare that they have no conflicts of interest with the contents of this article.

## Supporting information

Supplemental Material 4

Supplement al Material 5

## References

[1] Raimondi S., Maisonneuve P., Lowenfels A.B. (2009). Epidemiology of pancreatic cancer: an overview. Nat. Rev. Gastroenterol. Hepatol..

[2] Vogelzang N.J., Benowitz S.I., Adams S., Aghajanian C., Chang S.M., Dreyer Z.E. (2012). Clinical cancer advances 2011: annual report on progress against cancer from the American Society of Clinical Oncology. J. Clin. Oncol..

[3] Jonckheere N., Vasseur R., Van Seuningen I. (2017). The cornerstone K-RAS mutation in pancreatic adenocarcinoma: from cell signaling network, target genes, biological processes to therapeutic targeting. Crit. Rev. Oncol. Hematol..

[4] Pourshams A., Sepanlou S., Ikuta K. (2019). The global, regional, and national burden of pancreatic cancer and its attributable risk factors in 195 countries and territories, 1990–2017: a systematic analysis for the Global Burden of Disease Study 2017. Lancet Gastroenterol. Hepatol..

[5] Neoptolemos J.P., Kleeff J., Michl P., Costello E., Greenhalf W., Palmer D.H. (2018). Therapeutic developments in pancreatic cancer: current and future perspectives. Nat. Rev. Gastroenterol. Hepatol..

[6] Conroy T., Hammel P., Hebbar M., Ben Abdelghani M., Wei A.C., Raoul J.L. (2018). FOLFIRINOX or gemcitabine as adjuvant therapy for pancreatic cancer. N. Engl. J. Med..

[7] Rosenberg L. (1997). Treatment of pancreatic cancer. Promises and problems of tamoxifen, somatostatin analogs, and gemcitabine. Int. J. Pancreatol.

[8] Philip P.A., Mooney M., Jaffe D., Eckhardt G., Moore M., Meropol N. (2009). Consensus report of the national cancer institute clinical trials planning meeting on pancreas cancer treatment. J. Clin. Oncol..

[9] Torphy R.J., Fujiwara Y., Schulick R.D. (2020). Pancreatic cancer treatment: better, but a long way to go. Surg. Today.

[10] Hruban R.H., Goggins M., Parsons J., Kern S.E. (2000). Progression model for pancreatic cancer. Clin. Cancer Res..

[11] Feig C., Gopinathan A., Neesse A., Chan D.S., Cook N., Tuveson D.A. (2012). The pancreas cancer microenvironment. Clin. Cancer Res..

[12] Bailey P., Chang D.K., Nones K., Johns A.L., Patch A.M., Gingras M.C. (2016). Genomic analyses identify molecular subtypes of pancreatic cancer. Nature.

[13] Ryan D.P., Hong T.S., Bardeesy N. (2014). Pancreatic adenocarcinoma. N. Engl. J. Med..

[14] Grapin-Botton A. (2005). Ductal cells of the pancreas. Int. J. Biochem. Cell Biol.

[15] Scheffzek K., Ahmadian M.R., Kabsch W., Wiesmüller L., Lautwein A., Schmitz F., Wittinghofer A. (1997). The Ras-RasGAP complex: structural basis for GTPase activation and its loss in oncogenic Ras mutants. Science.

[16] Delpu Y., Hanoun N., Lulka H., Sicard F., Selves J., Buscail L. (2011). Genetic and epigenetic alterations in pancreatic carcinogenesis. Curr. Genomics.

[17] Stephen A.G., Esposito D., Bagni R.K., McCormick F. (2014). Dragging ras back in the ring. Cancer Cell.

[18] Bournet B., Buscail C., Muscari F., Cordelier P., Buscail L. (2016). Targeting KRAS for diagnosis, prognosis, and treatment of pancreatic cancer: hopes and realities. Eur. J. Cancer.

[19] Mann K.M., Ying H., Juan J., Jenkins N.A., Copeland N.G. (2016). KRAS-related proteins in pancreatic cancer. Pharmacol. Ther..

[20] Eser S., Schnieke A., Schneider G., Saur D. (2014). Oncogenic KRAS signalling in pancreatic cancer. Br. J. Cancer.

[21] Haigis K.M. (2017). KRAS alleles: the devil is in the detail. Trends Cancer.

[22] Skoulidis F., Li B.T., Dy G.K., Price T.J., Falchook G.S., Wolf J. (2021). Sotorasib for lung cancers with KRAS p.G12C mutation. N. Engl. J. Med..

[23] Fell J.B., Fischer J.P., Baer B.R., Blake J.F., Bouhana K., Briere D.M. (2020). Identification of the clinical development candidate MRTX849, a covalent KRASG12C inhibitor for the treatment of cancer. J. Med. Chem..

[24] Luo J. (2021). KRAS mutation in pancreatic cancer. Semin. Oncol..

[25] Wang X., Allen S., Blake J.F., Bowcut V., Briere D.M., Calinisan A. (2022). Identification of MRTX1133, a noncovalent, potent, and selective KRASG12D inhibitor. J. Med. Chem..

[26] Mao Z., Xiao H., Shen P., Yang Y., Xue J., Yang Y. (2022). KRAS(G12D) can be targeted by potent inhibitors via formation of salt bridge. Cell Discov.

[27] Akhave N.S., Biter A.B., Hong D.S. (2022). The next generation of KRAS targeting: reasons for excitement and concern. Mol. Cancer Ther..

[28] Nokin M.-J., Mira A., Patrucco E., Ricciuti B., Cousin S., Soubeyran I. (2024). RAS-ON inhibition overcomes clinical resistance to KRAS G12C-OFF covalent blockade. Nat. Commun..

[29] Conway J.R., Herrmann D., Evans T.J., Morton J.P., Timpson P. (2018). Combating pancreatic cancer with PI3K pathway inhibitors in the era of personalised medicine. Gut.

[30] Ricard-Blum S. (2011). The collagen family. Cold Spring Harb. Perspect. Biol..

[31] Begum A., Ewachiw T., Jung C., Huang A., Norberg K.J., Marchionni L. (2017). The extracellular matrix and focal adhesion kinase signaling regulate cancer stem cell function in pancreatic ductal adenocarcinoma. PLoS One.

[32] Lu J., Zhou S., Siech M., Habisch H., Seufferlein T., Bachem M.G. (2014). Pancreatic stellate cells promote hapto-migration of cancer cells through collagen I-mediated signalling pathway. Br. J. Cancer.

[33] Benesch M.G. (2022). High beta integrin expression is differentially associated with worsened pancreatic ductal adenocarcinoma outcomes. Am. J. Cancer Res..

[34] Kuo T.-C., Wu M.H., Yang S.H., Chen S.T., Hsu T.W., Jhuang J.Y. (2021). C1GALT1 high expression is associated with poor survival of patients with pancreatic ductal adenocarcinoma and promotes cell invasiveness through integrin αv. Oncogene.

[35] Gregori A., Bergonzini C., Capula M., Mantini G., Khojasteh-Leylakoohi F., Comandatore A. (2023). Prognostic significance of integrin subunit alpha 2 (ITGA2) and role of mechanical cues in resistance to gemcitabine in pancreatic ductal adenocarcinoma (PDAC). Cancers.

[36] Li J., Peng L., Chen Q., Ye Z., Zhao T., Hou S. (2022). Integrin β1 in pancreatic cancer: expressions, functions, and clinical implications. Cancers.

[37] Li Z., Lin P., Gao C., Peng C., Liu S., Gao H. (2016). Integrin β6 acts as an unfavorable prognostic indicator and promotes cellular malignant behaviors via ERK-ETS1 pathway in pancreatic ductal adenocarcinoma (PDAC). Tumor Biol..

[38] Erkan M., Michalski C.W., Rieder S., Reiser-Erkan C., Abiatari I., Kolb A. (2008). The activated stroma index is a novel and independent prognostic marker in pancreatic ductal adenocarcinoma. Clin. Gastroenterol. Hepatol..

[39] Petrova V., Annicchiarico-Petruzzelli M., Melino G., Amelio I. (2018). The hypoxic tumour microenvironment. Oncogenesis.

[40] Adhikari H., Counter C.M. (2018). Interrogating the protein interactomes of RAS isoforms identifies PIP5K1A as a KRAS-specific vulnerability. Nat. Commun..

[41] Ritchie C., Mack A., Harper L., Alfadhli A., Stork P.J.S., Nan X. (2017). Analysis of K-Ras interactions by biotin ligase tagging. Cancer Genomics Proteomics.

[42] Dekkers J.F., Alieva M., Wellens L.M., Ariese H.C.R., Jamieson P.R., Vonk A.M. (2019). High-resolution 3D imaging of fixed and cleared organoids. Nat. Protoc..

[43] Apweiler R., Bairoch A., Wu C.H., Barker W.C., Boeckmann B., Ferro S. (2004). UniProt: the universal protein knowledgebase. Nucleic Acids Res..

[44] Steinle J.J., Meininger C.J., Forough R., Wu G., Wu M.H., Granger H.J. (2002). Eph B4 receptor signaling mediates endothelial cell migration and proliferation via the phosphatidylinositol 3-kinase pathway. J. Biol. Chem..

[45] Gundry C., Marco S., Rainero E., Miller B., Dornier E., Mitchell L. (2017). Phosphorylation of Rab-coupling protein by LMTK3 controls Rab14-dependent EphA2 trafficking to promote cell:cell repulsion. Nat. Commun..

[46] Hill W., Hogan C. (2019). Normal epithelial cells trigger EphA2-dependent RasV12 cell repulsion at the single cell level. Small GTPases.

[47] Pasquale E.B. (2010). Eph receptors and ephrins in cancer: bidirectional signalling and beyond. Nat. Rev. Cancer.

[48] Boissier P., Chen J., Huynh-Do U. (2013). EphA2 signaling following endocytosis: role of Tiam1. Traffic.

[49] Hamaoka Y., Negishi M., Katoh H. (2016). EphA2 is a key effector of the MEK/ERK/RSK pathway regulating glioblastoma cell proliferation. Cell Signal.

[50] Finney A.C., Scott M.L., Reeves K.A., Wang D., Alfaidi M., Schwartz J.C. (2021). EphA2 signaling within integrin adhesions regulates fibrillar adhesion elongation and fibronectin deposition. Matrix Biol..

[51] Goldfinger L.E., Ptak C., Jeffery E.D., Shabanowitz J., Han J., Haling J.R. (2007). An experimentally derived database of candidate Ras-interacting proteins. J. Proteome Res..

[52] Béganton B., Coyaud E., Laurent E.M.N., Mangé A., Jacquemetton J., Le Romancer M. (2020). Proximal protein interaction landscape of RAS paralogs. Cancers (Basel).

[53] Che Y., Siprashvili Z., Kovalski J.R., Jiang T., Wozniak G., Elcavage L., Khavari P.A. (2019). KRAS regulation by small non-coding RNAs and SNARE proteins. Nat. Commun..

[54] Cheng D.K., Oni T.E., Thalappillil J.S., Park Y., Ting H.C., Alagesan B. (2021). Oncogenic KRAS engages an RSK1/NF1 pathway to inhibit wild-type RAS signaling in pancreatic cancer. Proc. Natl. Acad. Sci. U S A..

[55] Shankar S., Pitchiaya S., Malik R., Kothari V., Hosono Y., Yocum A.K. (2016). KRAS engages AGO2 to enhance cellular transformation. Cell Rep.

[56] Kovalski J.R., Bhaduri A., Zehnder A.M., Neela P.H., Che Y., Wozniak G.G., Khavari P.A. (2019). The functional proximal proteome of oncogenic ras includes mTORC2. Mol. Cell.

[57] Elowe S., Holland S.J., Kulkarni S., Pawson T. (2001). Downregulation of the Ras–mitogen-activated protein kinase pathway by the EphB2 receptor tyrosine kinase is required for ephrin-induced neurite retraction. Mol. Cell Biol..

[58] Macrae M., Neve R.M., Rodriguez-Viciana P., Haqq C., Yeh J., Chen C. (2005). A conditional feedback loop regulates Ras activity through EphA2. Cancer Cell.

[59] Chang Q., Jorgensen C., Pawson T., Hedley D.W. (2008). Effects of dasatinib on EphA2 receptor tyrosine kinase activity and downstream signalling in pancreatic cancer. Br. J. Cancer.

[60] Dechat T., Adam S.A., Taimen P., Shimi T., Goldman R.D. (2010). Nuclear lamins. Cold Spring Harb Perspect. Biol..

[61] (2013). SHC1 temporally regulates EGFR signaling. Cancer Discov..

[62] Shi W., Zhang G., Ma Z., Li L., Liu M., Qin L. (2021). Hyperactivation of HER2-SHCBP1-PLK1 axis promotes tumor cell mitosis and impairs trastuzumab sensitivity to gastric cancer. Nat. Commun..

[63] Yang P., Li W., Li X. (2022). SHC1 promotes lung cancer metastasis by interacting with EGFR. J. Oncol..

[64] Pawson T. (1995). Protein modules and signalling networks. Nature.

[65] Pawson T. (2004). Specificity in signal transduction: from Phosphotyrosine-SH2 domain interactions to complex cellular systems. Cell.

[66] Ahn R., Sabourin V., Ha J.R., Cory S., Maric G., Im Y.K. (2013). The ShcA PTB domain functions as a biological sensor of phosphotyrosine signaling during breast cancer progression. Cancer Res..

[67] Zheng Y., Zhang C., Croucher D.R., Soliman M.A., St-Denis N., Pasculescu A. (2013). Temporal regulation of EGF signalling networks by the scaffold protein Shc1. Nature.

[68] Below C.R., Kelly J., Brown A., Humphries J.D., Hutton C., Xu J. (2022). A microenvironment-inspired synthetic three-dimensional model for pancreatic ductal adenocarcinoma organoids. Nat. Mater..

[69] Koenig A., Mueller C., Hasel C., Adler G., Menke A. (2006). Collagen type I induces disruption of E-cadherin-mediated cell-cell contacts and promotes proliferation of pancreatic carcinoma cells. Cancer Res..

[70] Giaginis C., Tsourouflis G., Zizi-Serbetzoglou A., Kouraklis G., Chatzopoulou E., Dimakopoulou K., Theocharis S.E. (2010). Clinical significance of ephrin (eph)-A1, -A2, -a4, -a5 and -a7 receptors in pancreatic ductal adenocarcinoma. Pathol. Oncol. Res..

[71] Grzesiak J.J., Tran Cao H.S., Burton D.W., Kaushal S., Vargas F., Clopton P. (2011). Knockdown of the beta(1) integrin subunit reduces primary tumor growth and inhibits pancreatic cancer metastasis. Int. J. Cancer.

[72] Lu H., Liu C., Velazquez R., Wang H., Dunkl L.M., Kazic-Legueux M. (2019). SHP2 inhibition overcomes RTK-mediated pathway reactivation in KRAS-mutant tumors treated with MEK inhibitors. Mol. Cancer Ther..

[73] Liu L., Wang S., Cen C., Peng S., Chen Y., Li X. (2019). Identification of differentially expressed genes in pancreatic ductal adenocarcinoma and normal pancreatic tissues based on microarray datasets. Mol. Med. Rep..

[74] Humphrey E.S., Su S.P., Nagrial A.M., Hochgräfe F., Pajic M., Lehrbach G.M. (2016). Resolution of novel pancreatic ductal adenocarcinoma subtypes by global phosphotyrosine profiling. Mol. Cell Proteomics.

[75] Lu Z., Zhang Y., Li Z., Yu S., Zhao G., Li M. (2012). Overexpression of the B-type Eph and ephrin genes correlates with progression and pain in human pancreatic cancer. Oncol. Lett..

[76] Wang H., Hou W., Perera A., Bettler C., Beach J.R., Ding X. (2021). Targeting EphA2 suppresses hepatocellular carcinoma initiation and progression by dual inhibition of JAK1/STAT3 and AKT signaling. Cell Rep..

[77] Chen Y.H., Lv H., Shen N., Wang X.M., Tang S., Xiong B. (2020). EPHA2 feedback activation limits the response to PDEδ inhibition in KRAS-dependent cancer cells. Acta Pharmacol. Sin.

[78] Porazinski S., de Navascués J., Yako Y., Hill W., Jones M.R., Maddison R. (2016). EphA2 drives the segregation of ras-transformed epithelial cells from normal neighbors. Curr. Biol..

[79] Hill W., Zaragkoulias A., Salvador-Barbero B., Parfitt G.J., Alatsatianos M., Padilha A. (2021). EPHA2-dependent outcompetition of KRASG12D mutant cells by wild-type neighbors in the adult pancreas. Curr. Biol..

[80] Wary K.K., Mainiero F., Isakoff S.J., Marcantonio E.E., Giancotti F.G. (1996). The adaptor protein Shc couples a class of integrins to the control of cell cycle progression. Cell.

[81] Yang X., Ma X., Zhao T., Croucher D.R., Nguyen E.V., Clark K.C. (2024). Feed-forward stimulation of CAMK2 by the oncogenic pseudokinase PEAK1 generates a therapeutically ‘actionable’ signalling axis in triple negative breast cancer. bioRxiv.

[82] Tyanova S., Temu T., Cox J. (2016). The MaxQuant computational platform for mass spectrometry-based shotgun proteomics. Nat. Protoc..

[83] Teo G., Liu G., Zhang J., Nesvizhskii A.I., Gingras A.C., Choi H. (2014). SAINTexpress: improvements and additional features in significance analysis of INTeractome software. J. Proteomics.

[84] Perez-Riverol Y., Bai J., Bandla C., García-Seisdedos D., Hewapathirana S., Kamatchinathan S. (2021). The PRIDE database resources in 2022: a hub for mass spectrometry-based proteomics evidences. Nucleic Acids Res..

