# Supplementary material for: A ternary association of KRas, EphA2, and integrins controls PDAC cell behavior
Source: J Biol Chem. 2026 Aug 10;302(10):113421. doi: 10.1016/j.jbc.2026.113421 (PMC13563243; doi:10.1016/j.jbc.2026.113421)
Supplement: Supplementary file 1 [file mmc1.pdf]

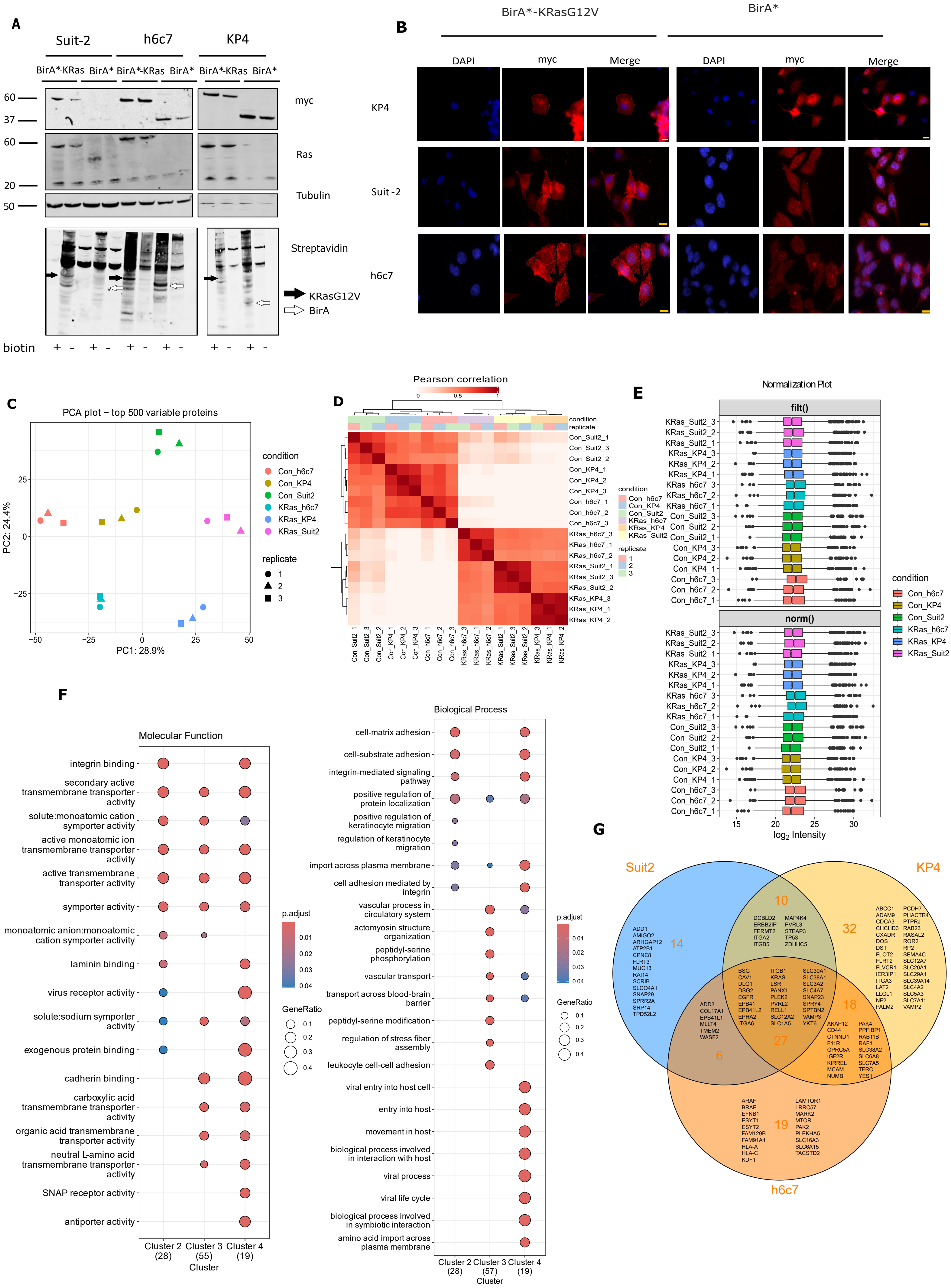

A
Suit-2
h6c7
KP4
BirA*-KRas
BirA*
60
37
20
50
myc
Ras
Tubulin
Streptavidin
KRasG12V
BirA
biotin
+ - + - + - + - + - + -
B
BirA*-KRasG12V
BirA*
DAPI
myc
Merge
KP4
Suit -2
h6c7
C
PCA plot – top 500 variable proteins
PC1: 28.9%
PC2: 24.4%
condition
Con_h6c7
Con_KP4
Con_Suit2
KRas_h6c7
KRas_KP4
KRas_Suit2
replicate
1
2
3
D
Pearson correlation
condition
replicate
Con_Suit2_1
Con_Suit2_3
Con_Suit2_2
Con_KP4_1
Con_KP4_2
Con_KP4_3
Con_h6c7_1
Con_h6c7_2
Con_h6c7_3
KRas_h6c7_3
KRas_h6c7_1
KRas_h6c7_2
KRas_Suit2_1
KRas_Suit2_3
KRas_Suit2_2
KRas_KP4_3
KRas_KP4_1
KRas_KP4_2
E
Normalization Plot
filt()
norm()
log2 Intensity
condition
Con_h6c7
Con_KP4
Con_Suit2
KRas_h6c7
KRas_KP4
KRas_Suit2
F
Molecular Function
integrin binding
secondary active transmembrane transporter activity
solute:monoatomic cation symporter activity
active monoatomic ion transmembrane transporter activity
active transmembrane transporter activity
symporter activity
monoatomic anion:monoatomic cation symporter activity
laminin binding
virus receptor activity
solute:sodium symporter activity
exogenous protein binding
cadherin binding
carboxylic acid transmembrane transporter activity
organic acid transmembrane transporter activity
neutral L-amino acid transmembrane transporter activity
SNAP receptor activity
antiporter activity
Cluster 2 (28)
Cluster 3 (55)
Cluster 4 (19)
Cluster
p.adjust
GeneRatio
Biological Process
cell-matrix adhesion
cell-substrate adhesion
integrin-mediated signaling pathway
positive regulation of protein localization
positive regulation of keratinocyte migration
regulation of keratinocyte migration
import across plasma membrane
cell adhesion mediated by integrin
vascular process in circulatory system
actomyosin structure organization
peptidyl-serine phosphorylation
vascular transport
transport across blood-brain barrier
peptidyl-serine modification
regulation of stress fiber assembly
leukocyte cell-cell adhesion
viral entry into host cell
entry into host
movement in host
biological process involved in interaction with host
viral process
viral life cycle
biological process involved in symbiotic interaction
amino acid import across plasma membrane
Cluster 2 (28)
Cluster 3 (57)
Cluster 4 (19)
Cluster
G
Suit2
KP4
h6c7
14
10
32
18
27
6
19
