# Supplementary material for: A ternary association of KRas, EphA2, and integrins controls PDAC cell behavior
Source: J Biol Chem. 2026 Aug 10;302(10):113421. doi: 10.1016/j.jbc.2026.113421 (PMC13563243; doi:10.1016/j.jbc.2026.113421)
Supplement: Supplementary file 2 [file mmc2.pdf]

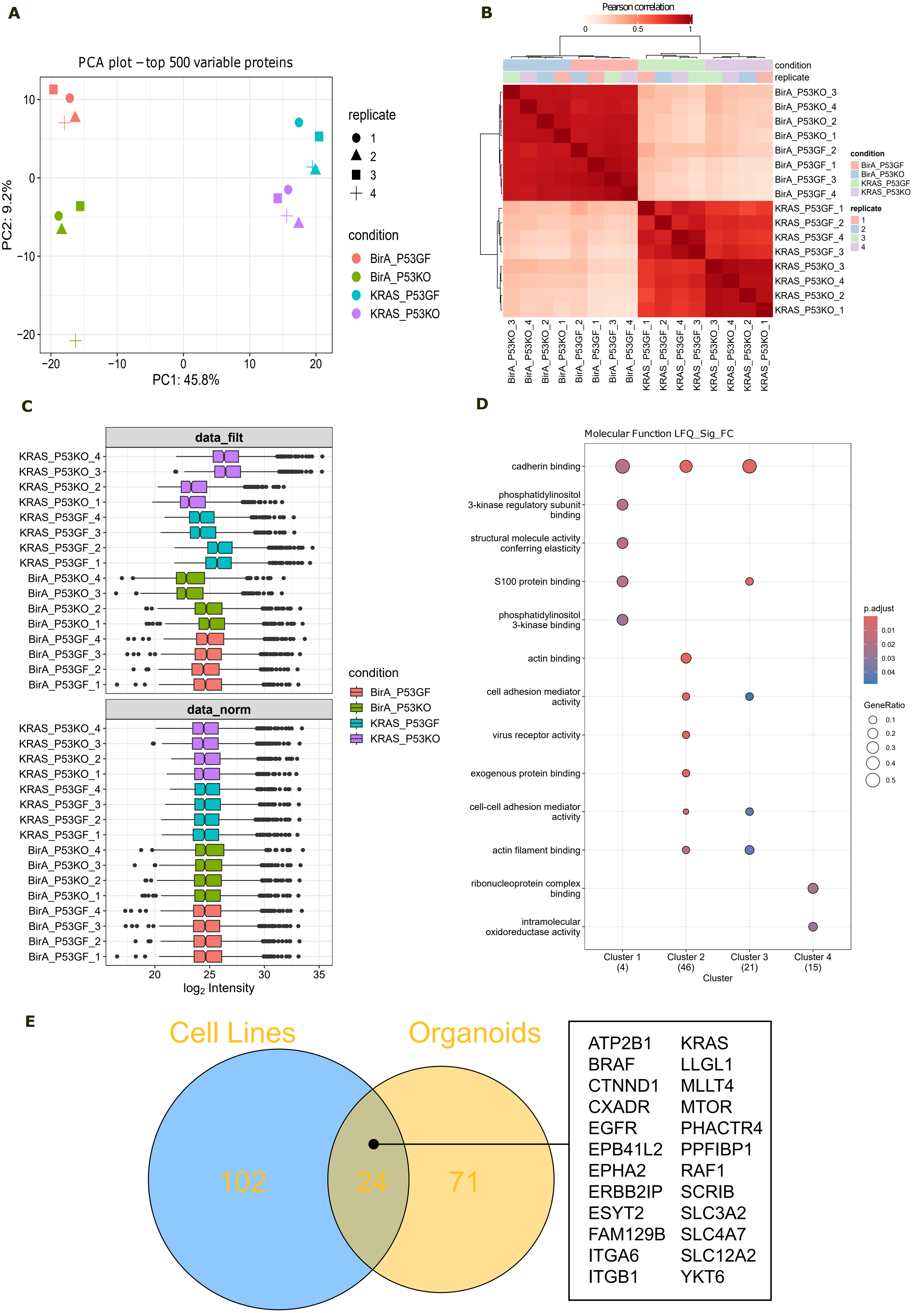

A
PCA plot – top 500 variable proteins
PC2: 9.2%
PC1: 45.8%
replicate
1
2
3
4
condition
BirA_P53GF
BirA_P53KO
KRAS_P53GF
KRAS_P53KO
B
Pearson correlation
0
0.5
1
condition
replicate
BirA_P53KO_3
BirA_P53KO_4
BirA_P53KO_2
BirA_P53KO_1
BirA_P53GF_2
BirA_P53GF_1
BirA_P53GF_3
BirA_P53GF_4
KRAS_P53GF_1
KRAS_P53GF_2
KRAS_P53GF_4
KRAS_P53GF_3
KRAS_P53KO_3
KRAS_P53KO_4
KRAS_P53KO_2
KRAS_P53KO_1
C
data_filt
data_norm
log2 Intensity
condition
BirA_P53GF
BirA_P53KO
KRAS_P53GF
KRAS_P53KO
D
Molecular Function LFQ_Sig_FC
cadherin binding
phosphatidylinositol 3-kinase regulatory subunit binding
structural molecule activity conferring elasticity
S100 protein binding
phosphatidylinositol 3-kinase binding
actin binding
cell adhesion mediator activity
virus receptor activity
exogenous protein binding
cell-cell adhesion mediator activity
actin filament binding
ribonucleoprotein complex binding
intramolecular oxidoreductase activity
Cluster 1 (4)
Cluster 2 (46)
Cluster 3 (21)
Cluster 4 (15)
Cluster
p.adjust
0.01
0.02
0.03
0.04
GeneRatio
0.1
0.2
0.3
0.4
0.5
E
Cell Lines
Organoids
102
24
71
ATP2B1
BRAF
CTNND1
CXADR
EGFR
EPB41L2
EPHA2
ERBB2IP
ESYT2
FAM129B
ITGA6
ITGB1
KRAS
LLGL1
MLLT4
MTOR
PHACTR4
PPFIBP1
RAF1
SCRIB
SLC3A2
SLC4A7
SLC12A2
YKT6
