# Supplementary material for: A ternary association of KRas, EphA2, and integrins controls PDAC cell behavior
Source: J Biol Chem. 2026 Aug 10;302(10):113421. doi: 10.1016/j.jbc.2026.113421 (PMC13563243; doi:10.1016/j.jbc.2026.113421)
Supplement: Supplementary file 3 [file mmc3.pdf]

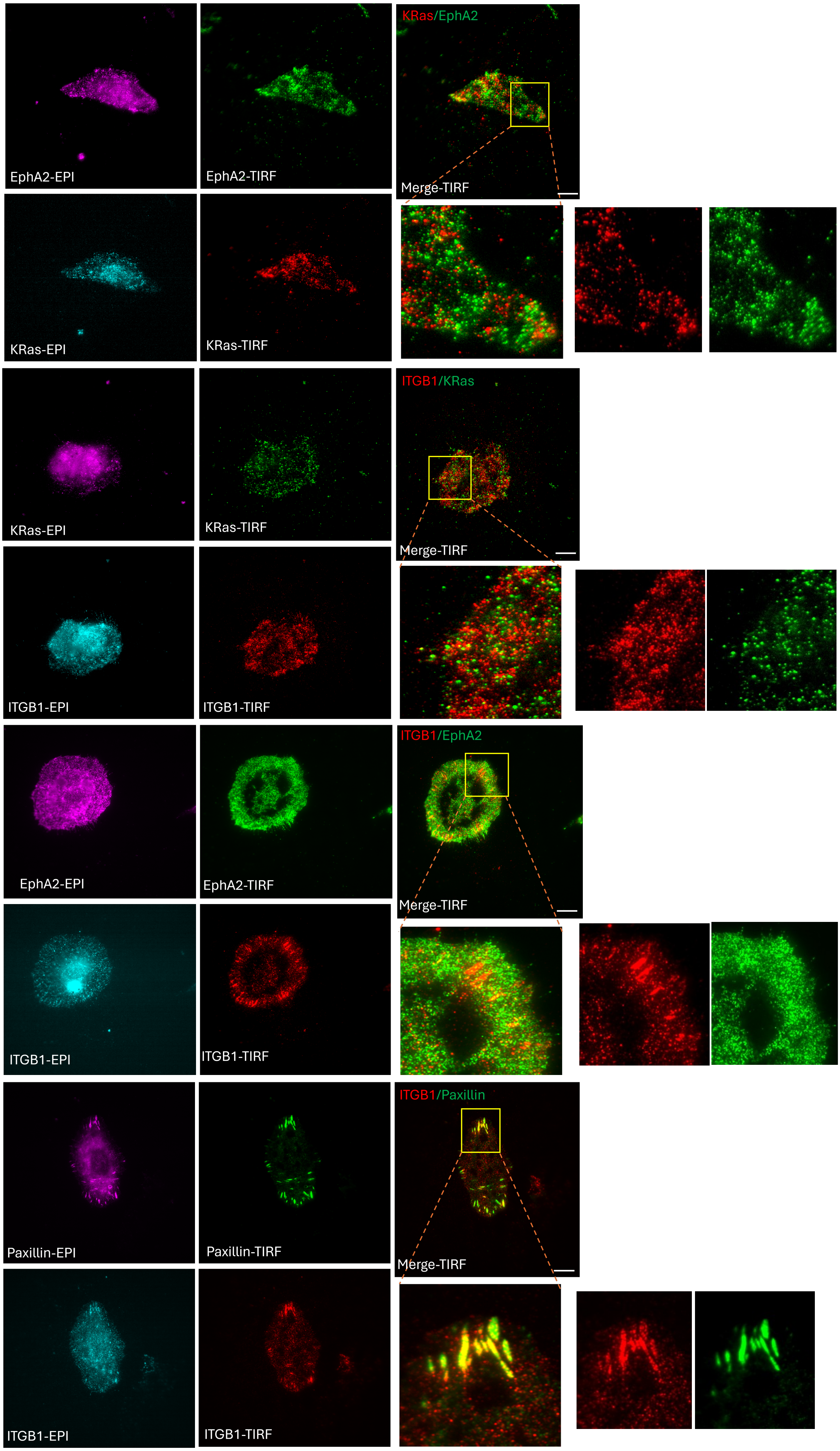

EphA2-EPI
EphA2-TIRF
KRas/EphA2
Merge-TIRF
KRas-EPI
KRas-TIRF
KRas-EPI
KRas-TIRF
ITGB1/KRas
Merge-TIRF
ITGB1-EPI
ITGB1-TIRF
EphA2-EPI
EphA2-TIRF
ITGB1/EphA2
Merge-TIRF
ITGB1-EPI
ITGB1-TIRF
Paxillin-EPI
Paxillin-TIRF
ITGB1/Paxillin
Merge-TIRF
ITGB1-EPI
ITGB1-TIRF
