# Supplementary material for: A ternary association of KRas, EphA2, and integrins controls PDAC cell behavior
Source: J Biol Chem. 2026 Aug 10;302(10):113421. doi: 10.1016/j.jbc.2026.113421 (PMC13563243; doi:10.1016/j.jbc.2026.113421)
Supplement: Supplementary file 5 [file mmc5.docx]

**Supplementary Figures**

**Supplementary Figure 1. Mass spectrometry analysis of KRas proximity interactome in human pancreatic cancer cell lines. A.** Immunofluorescence images for pCDH-EF1-tagBFP-T2A-myc-Bir-A* and pCDH-EF1-tagBFP-T2A-myc-Bir-A*-KRas^G12V^-expressing stable cell lines. Staining was performed using an anti-myc tag antibody and visualised with Alexa Fluor 488 conjugated antibody (red). Nuclei were stained with DAPI (blue). Images are representative of staining seen across samples. Scale bar, 10µM. **B.** PDAC cell lines with stable expression of BirA*-KRas^G12V^ or BirA* proteins were subjected to western blot analysis following 24 hours of incubation with or without biotin (50µM) and subsequent lysis. Indicated antibodies were used to detect protein expression. Fluorescently labelled streptavidin antibody was used to detect biotinylated proteins. **C-E.** Affinity-purified proteins from the BioID experiment of control and myc-BirA*-KRas^G12V^-expressing KP4, Suit-2 and h6c7 cells were subjected to LC-MS/MS analysis and raw data were analysed using MaxQuant. **C.** PCA was performed on each replicate of BirA*-KRas^G12V^ and control groups, and principal components 1 and 2 were plotted. PC1, principal component 1; PC2, principal component 2. **D.** Total LFQ intensity values for proteins identified in each repeat of BirA* and KRas-expressing cells were compared in a pairwise comparison and Pearson correlation values were calculated. Pearson correlation plot showing differentially expressed proteins obtained in the pairwise comparison of KRas and BirA*-expressing cell lines. Significant proteins were enriched with a BFDR score ≤0.05. **E.** Normalisation plot of the filtered or original intensity values identified in each replicate. **F.** Protein lists from SAINT analysis were imported into “R” and clusterProfiler package was used to perform gene ontology enrichment analysis. The chart represents significantly enriched proteins in the molecular functions and biological processes category. The dot size represents the gene ratio for the associated GO term, and the colour represents the adjusted p-value. **G**. The Venn diagram represents the common and distinct interactors identified as significant protein hits from KP4, Suit-2 and h6c7 cell lines following mass spectrometry and bioinformatics analysis.

**Supplementary Figure 2. Mass spectrometry analysis of KRas proximity interactome in PDAC organoids.** Affinity-purified proteins from the BioID experiment of control and KRas-expressing PDAC organoids (with knockout or mutant P53) were subjected to LC-MS/MS analysis and raw data were analysed using MaxQuant. **A.** PCA was performed on each replicate and principal components 1 and 2 were plotted. PC1, principal component 1; PC2, principal component 2. **B.** Total LFQ intensity values for proteins identified in each replicate were compared pairwise and Pearson correlation values were plotted. **C.** Normalisation plot of the filtered or original intensity values identified in each replicate. **D.** Protein lists from knockout or mutant PDAC models were imported into “R” and the clusterProfiler package was used to perform gene ontology enrichment analysis. The chart represents the molecular functions category of the enriched proteins. The dot size represents the gene ratio for the associated GO term and the colour represents the adjusted p-value. **E.** Following MaxQuant analysis on the mass spectrometry data obtained for the cell lines and organoid models, the significant protein hits were subjected to comparative analysis to identify common and distinct interactors of KRas.

**Supplementary Figure 3. Colocalisation of KRas with EphA2 and integrin β1.** Serum-starved U2OS cells were sparsely seeded on fibronectin-coated glass bottom dishes and fixed with 4% (v/v) PFA. Staining was performed using indicated antibodies and visualised with Alexa Fluor 488 and Alexa Fluor-594 conjugated antibodies in TIRF mode (green and red respectively) at 90-90 angles for incidence and reflection of light. The ROI was selected to show the colocalisation of stained proteins in detail. Images are representative of staining seen across samples. Scale bar, 25µm.

**Supplementary Figure 4. Mechanism of interaction between KRas, EphA2 and integrin β1.** KP4 cells were targeted by KRas-specific or scrambled siRNA and protein expression was examined by western blotting 48hr post-transfection. The graph shows the expression of proteins in arbitrary units following normalisation to tubulin (Dm1A antibody) and the comparison between the knockdown group vs the control group (n=2). Error bars represent standard deviation.
