# Supplementary material for: A ternary association of KRas, EphA2, and integrins controls PDAC cell behavior
Source: J Biol Chem. 2026 Aug 10;302(10):113421. doi: 10.1016/j.jbc.2026.113421 (PMC13563243; doi:10.1016/j.jbc.2026.113421)
Supplement: Supplementary file 6 [file mmc6.docx]

**Supplementary Table 1**

**Primary and secondary antibodies used in this study**

| **Target** | | **Application** | **Dilution** | **Source species** | **Company, Catalogue Number** |
| --- | --- | --- | --- | --- | --- |
| **Primary Antibodies:** | |  |  |  |  |
| Ras (D2C1) | | WB | 1:1000 | Rabbit mAb | Cell Signalling Technology (8955) |
| KRas (9.13) | | IF | 1:100 | Mouse mAb | Invitrogen (415700) |
|  | |  |  |  |  |
| Integrin β1 | | WB | 1:1000 | Rabbit mAb | Abcam (ab52971) |
| Integrin β1 | | IF | 1:1000 | Rat | 9EG7 |
| EphA2 (D4A2)  EphA2 (8B6) | | WB  /IF  IF | 1:1000/1:400  1:400 | Rabbit mAb  Mouse mAb | Cell Signalling Technology  (6997)  12927 |
| Myc-tag (9B11) | | WB  IF | 1:1000  1:300 | Mouse mAb | Cell Signalling Technology (2276) |
| Tubulin | | WB | 1:1000 | Mouse | Sigma-Aldrich (T9026) |
| AKT (c67e7) | | WB | 1:1000 | Rabbit mAb | Cell Signalling Technology (4691) |
| Phospho-AKT (Ser473) | | WB | 1:1000 | Rabbit mAb | Cell Signalling Technology (4060) |
| P44/42-MAPK (ERK1/2) | | WB | 1:1000 | Mouse mAb | Cell Signalling Technology (4695) |
| P44/42 MAPK (ERK1/2) | | WB | 1:1000 | Rabbit | Cell Signalling Technology (4377) |
| Shc1 | | WB  IF | 1:1000  1:500 | Rabbit mAb | BD-Biosciences |
| Paxillin | | IF | 1:1000 | Rabbit | GTX125891 |
| **Secondary Antibodies** |  |  |  |  |  |
| Anti-mouse IgG DyLightTM 680 Conjugate | | WB | 1:10000 | Goat | Invitrogen (A21058) |
| Anti-mouse IgG DyLightTM 800 Conjugate | | WB | 1:10000 | Goat | Invitrogen (A21109) |
| Anti-rabbit IgG DyLightTM 680 Conjugate | | WB | 1:10000 | Goat | Invitrogen (A32730) |
| Anti-rabbit IgG DyLightTM 800 Conjugate | | WB | 1:10000 | Donkey | Invitrogen (A32735) |
| Anti-Mouse IgG Alexa Fluor 488 | | IF | 1:500 | Donkey | Invitrogen |
| Anti-Rabbit IgG Alexa Fluor 594 | | IF | 1:500 | Donkey | Invitrogen |
| Anti-Rat IgG Alexa Fluor 647 | | IF | 1:500 | Donkey | Invitrogen |

WB, western blotting; IF, Immunofluorescence
