# Supplementary material for: A ternary association of KRas, EphA2, and integrins controls PDAC cell behavior
Source: J Biol Chem. 2026 Aug 10;302(10):113421. doi: 10.1016/j.jbc.2026.113421 (PMC13563243; doi:10.1016/j.jbc.2026.113421)
Supplement: Supplementary file 7 [file mmc7.docx]

**Supplementary Table 2**

**siRNAs used in this study**

| Gene Name | Gene ID | Catalogue No | siRNA | Company |
| --- | --- | --- | --- | --- |
| EphA2 | 1969 | L-003116-00-0005 | ON-TARGETplus Human siRNA SMARTpool | Dharmacon horizon discovery |
| EphA2 | 1969 | CTM-809756 | Custom EPHA2 duplex.  Sense: 5' GGGACCUGAUGCAGAACAUUU 3'  Antisense: 5' AUGUUCUGCAUCAGGUCCCUU 3' | Dharmacon horizon discovery |
| KRas | 3845 | L-005069-00-0005 | ON-TARGETplus Human siRNA SMARTpool | Dharmacon horizon discovery |
| Integrin β1 | 3688 | L-004506-00-0005 | ON-TARGETplus Human siRNA SMARTpool | Dharmacon horizon discovery |
| Control | ---- | D-001810-01-20 | ON-TARGETplus Non-targeting Control siRNAs | Dharmacon horizon discovery |
